# Single-nucleus RNA sequencing of human pancreatic islets identifies novel gene sets and distinguishes β-cell subpopulations with dynamic transcriptome profiles

**DOI:** 10.1186/s13073-023-01179-2

**Published:** 2023-05-01

**Authors:** Randy B. Kang, Yansui Li, Carolina Rosselot, Tuo Zhang, Mustafa Siddiq, Prashant Rajbhandari, Andrew F. Stewart, Donald K. Scott, Adolfo Garcia-Ocana, Geming Lu

**Affiliations:** 1 Diabetes, Obesity and Metabolism Institute, and Division of Endocrinology, Diabetes and Bone Diseases, Icahn School of Medicine at Mount Sinai, New York, NY 10029 USA; 2 Present address: Department of Molecular and Cellular Endocrinology, Arthur Riggs Diabetes and Metabolism Research Institute, Beckman Research Institute, City of Hope, 1500 East Duarte Road, Duarte, CA 91010 USA; 3 Genomics Resources Core Facility, Weill Cornell Medicine, New York, NY 10065 USA; 4 Mindich Child Health and Development Institute, Icahn School of Medicine at Mount Sinai, New York, NY 10029 USA; 5 Department of Pharmacological Sciences and Institute for Systems Biomedicine, Icahn School of Medicine at Mount Sinai, New York, NY 10029 USA

**Keywords:** Diabetes, Human islet, Human pancreatic beta cell, Single-cell RNA sequencing, Single-nucleus RNA sequencing, Human islet graft

## Abstract

**Background:**

Single-cell RNA sequencing (scRNA-seq) provides valuable insights into human islet cell types and their corresponding stable gene expression profiles. However, this approach requires cell dissociation that complicates its utility in vivo. On the other hand, single-nucleus RNA sequencing (snRNA-seq) has compatibility with frozen samples, elimination of dissociation-induced transcriptional stress responses, and affords enhanced information from intronic sequences that can be leveraged to identify pre-mRNA transcripts.

**Methods:**

We obtained nuclear preparations from fresh human islet cells and generated snRNA-seq datasets. We compared these datasets to scRNA-seq output obtained from human islet cells from the same donor. We employed snRNA-seq to obtain the transcriptomic profile of human islets engrafted in immunodeficient mice. In both analyses, we included the intronic reads in the snRNA-seq data with the GRCh38-2020-A library.

**Results:**

First, snRNA-seq analysis shows that the top four differentially and selectively expressed genes in human islet endocrine cells in vitro and in vivo are not the canonical genes but a new set of non-canonical gene markers including *ZNF385D*, *TRPM3*, *LRFN2*, *PLUT* (β-cells); *PTPRT*, *FAP*, *PDK4*, *LOXL4* (α-cells); *LRFN5*, *ADARB2*, *ERBB4*, *KCNT2* (δ-cells); and *CACNA2D3*, *THSD7A*, *CNTNAP5*, *RBFOX3* (γ-cells). Second, by integrating information from scRNA-seq and snRNA-seq of human islet cells, we distinguish three β-cell sub-clusters: an *INS* pre-mRNA cluster (β3), an intermediate *INS* mRNA cluster (β2), and an *INS* mRNA-rich cluster (β1). These display distinct gene expression patterns representing different biological dynamic states both in vitro and in vivo. Interestingly, the INS mRNA-rich cluster (β1) becomes the predominant sub-cluster in vivo.

**Conclusions:**

In summary, snRNA-seq and pre-mRNA analysis of human islet cells can accurately identify human islet cell populations, subpopulations, and their dynamic transcriptome profile in vivo.

**Supplementary Information:**

The online version contains supplementary material available at 10.1186/s13073-023-01179-2.

## Background

Diabetes results from deficiency of functional pancreatic β-cells [1, 2]. Detailed characterization of the transcriptional programs in islet cells in health and disease will help to identify therapeutic targets to treat diabetes [3]. The recent, and now widely used, application of single-cell RNA sequencing (scRNA-seq) on human islets from healthy donors and patients with diabetes is providing a wealth of data regarding islet cell populations and their established transcriptome profile [4–10]. scRNA-seq of dispersed cells from human islets or pancreas tissue represents an obvious advance over bulk RNA sequencing of whole islets or tissue, and a clear improvement over bulk transcriptomic analysis of sorted islet cell subtypes, both of which require mechanical and enzymatic cell disruption and inevitable cellular stress. scRNA-seq also requires mechanical and enzymatic cell dissociation with unavoidable adverse biological consequences on islet cell subtypes. Further, scRNA-seq typically focusses on exon reads, which provides limited or no information on the pre-mRNA status of the corresponding genes, since most of the mRNA analyzed is mature, stored mRNA [4].scRNA-seq on human islet cells has identified genes associated with type 1 (T1D) and type 2 (T2D) diabetes [5–7], genes important for islet cell development and maturation [8, 9], for islet dysfunction and dedifferentiation [10, 11], for aging [12], and genes involved in the transdifferentiation among islet cells [13, 14]. These studies typically use “canonical” gene sets to annotate different islet cell populations, and new gene sets are continuously identified that more precisely define islet cell subtypes as they passage from development, stem cell differentiation through maturation [15]. scRNA-seq has also confirmed the presence of heterogeneity among human β-cells, defining several β-cell subtypes with different gene profiles [16, 17]. Although these are remarkable advances, it remains true that these scRNA-seq studies have been mostly performed using isolated human islets cultured in vitro, which may not reflect actual in vivo biology. Further, scRNA-seq analysis of human cadaveric pancreas biopsies or human islet grafts in mice requires mechanical and enzymatic cell dissociation, which results in low cellular yields, causes non-physiologic cellular stress, eliminates specific cell subpopulations sensitive to these stresses, and thus represents small subpopulations of islet cells that survived the vicissitudes of organ donation, islet isolation, and islet cell dispersion. Thus, scRNA-seq datasets may not reflect the complete universe of the normal islet cell population [18–22].

In contrast, single-nucleus RNA sequencing (snRNA-seq) uses isolated nuclei, has substantial advantages including reduced dissociation bias, compatibility with frozen samples, and elimination of dissociation-induced transcriptional stress responses, and uncovers mostly unspliced pre-mRNAs containing introns, as well as exons. Combining exonic and intronic sequences reveals important information on the pre-mRNA transcriptome status of a cell at a given moment in time [23, 24]. This approach should also be adaptable to analysis of pre-mRNA transcriptome profiles of human islet cells in vivo in previously fixed or frozen samples, as well as in human islet grafts from mice. Pioneer work by Basile et al. recently reported a side-by-side comparison of snRNA-seq and scRNA-seq of human islet cells to validate the robustness of the former as an alternative sequencing strategy especially when scRNA-seq is not ideal [22]. They observed virtually complete concordance in identifying human islet cell types and gene proportions as well as a strong association of global and islet cell type gene signatures between scRNA-seq and snRNA-seq. However, these studies did not take advantage of the direct comparison of exonic mature mRNA reads (scRNA-seq) vs. exonic plus intronic reads (pre-mRNA, snRNA-seq) to infer the real-time global pre-mRNA transcriptome status of the cell. This is in part due to the absence of human islet cell annotation references that include intronic data, and the absence of adequate gene sets to identify human islet cells using snRNA-seq analysis. In addition, these studies did not identify different β-cell subpopulations using snRNA-seq datasets.

In this study, we addressed these latter issues by directly comparing scRNA-seq and snRNA-seq analysis of human islet cells to determine whether (1) they provide similar results on human islet cell populations; (2) intron plus exon reads in the snRNA-seq analysis provide additional information that further defines islet cell populations; (3) new gene sets can be defined in the snRNA-seq analysis that more accurately portray islet cell populations; (4) β-cell heterogeneity can be defined using snRNA-seq analysis; and (5) the snRNA-seq approach can enrich analysis of transcriptome profiles in human islets in vivo. Reassuringly, the results indicate that scRNA-seq and snRNA-seq using exon or combined intron plus exon reads, respectively, are interchangeable to identify human islet cell populations both in vitro and in vivo. However, the results also make the important point that at the pre-mRNA level, canonical endocrine cell genes are not the top highly and selectively expressed genes in these islet endocrine cells. Conversely, these studies reveal new, non-canonical gene sets that accurately identify endocrine cell types in the snRNA-seq analysis of human islets that can be used in in vitro as well as in vivo human islet studies. Finally, integrating datasets from both scRNA-seq and snRNA-seq analyses, we distinguish three different β-cell subpopulations reflecting differences in their *INS* mRNA transcriptional stage. These differences imply distinct biological functions according to gene set enrichment analysis (GSEA). Overall, this study supports the use of snRNA-seq technology and pre-mRNA analysis as a tool for deciphering human islet cell populations and subpopulations and their distinct biological functions in health and disease.

## Methods

### Human islet samples

Adult human pancreatic islets from seven brain-dead donors were generated by Prodo Laboratories (Aliso Viejo, CA) according to the standard procedures [25]. Islets were harvested from pancreata from deceased organ donors without any identifying information and with informed consent properly and legally secured, and Western Institutional Review Board (WIRB) approval. The average donor age was 38 ± 5 and 71% of them were male donors. Complete donor demographic information is provided in Additional file 1: Table S1. Human islets were procured in serum-free medium, centrifuged, washed, and then cultured in complete RPMI medium (5 mM glucose) and non-adhesive culture plates (3000–5000 human islets equivalents [IEQs] 1 IEQ = 150 μm diameter islet/plate) in 5% CO_2_ at 37 °C overnight before initiation of the studies. Human pancreatic islets from three donors (*N* = 3) were used for the in vitro studies (detailed below) and human islets from four donors were used in human islet transplant experiments in immunosuppressed mice (detailed below).

### Human islet cells and nuclei processing

Human islets from three different donors (3000 IEQs/donor) (Additional file 1: Table S1) were collected, washed twice with PBS (Ca2 + /Mg2 + free), and then centrifuged at 300 rpm for 3 min. After removing the PBS, 200 µl pre-warmed Accutase (cat# 25–058-CL, Corning) were added and the islets were incubated at 37 °C for 10 min. Then, complete RPMI medium was added to the tubes, the samples were centrifuged at 1000 rpm for 3 min, and the pellet washed with PBS (Ca2 + /Mg2 + free). Half of the cells were resuspended in binding buffer (cat# 130–090-101, Miltenyi Biotec) with dead cell removal beads, incubated for 15 min at room temperature and applied onto the dead cell removal column (cat # 130–042-401, Miltenyi Biotec), which was attached to the MACS separator. Subsequently, the effluent was collected, centrifuged, and resuspended with 200 µl 2% BSA and 200 U/ml RNase inhibitor in PBS. The cells were then mixed with AOPI (Cat# CS2-0106, Nexcelon Bioscience) at 1:1 ratio and the cell concentration measured with the Countess 3 Automated Cell Counter (Thermo-Fisher). The other half of the cells was homogenized with a pestle, and their nuclei were isolated with the Minute™ single nucleus isolation kit for tissue/cells (Cat# SN-047, Invent Biotechnologies, INC). Briefly, cells were resuspended in 600 µl cold lysis buffer, incubated on ice for 10 min and then transferred into a filter with a collection tube. The tubes were then centrifuged at 600 × *g* for 5 min, the supernatants removed, and the pellets resuspended in 500 µl cold washing buffer. After centrifugation at 500 × *g* for 5 min, the supernatants were removed, and the nuclei pellet resuspended with 55 µl 2% BSA and 200 U/ml RNase inhibitor in PBS. Nuclei were then mixed with AOPI at 1:1 ratio and the nuclei concentration measured with the Countess 3 Automated Cell Counter. After this, nuclei samples were processed in an identical way as to the cell samples.

### Human islet transplantation into RAG-1^−/−^ immunodeficient mice

One thousand human IEQs from four different donors (Additional file 1: Table S1) were transplanted into the renal sub-capsular space of 4–5-month-old euglycemic RAG1^*−*/*−*^ mice as described previously in detail [26, 27], and the animals were followed for 3 months. At the end of the follow-up period, mice were sacrificed by CO_2_ inhalation. Human islet grafts harvested 3 months after transplantation were washed twice with PBS (Ca2 + /Mg2 + free) and centrifuged at 300 rpm for 3 min. Nuclei were isolated as indicated above. Animal studies and procedures were performed with the approval of and in accordance with guidelines established by the Institutional Animal Care and Use Committee of the Icahn School of Medicine at Mount Sinai (IACUC #2015–0107).

### Single-cell and single-nucleus RNA sequencing, alignment, and matrix generation

Cells and nuclei samples were prepared according to the 10X Genomics Single Cell 3’ V3.1 Reagent Kit protocol, processed with 10X Genomic Chromium Controller for partitioning and barcoding, followed by the cDNA library generation. The total cell concentration was analyzed by Countess 3, then sequenced by NovaSeq 6000 System (Illumina) at the Weill Cornell Medicine, Genomics and Epigenomics Core. FASTQ files were aligned with Cell Ranger V.6.1.1 with Single Cell 3’ V3 chemistry on the 10X Cloud’s pipeline. In the analysis, we included the intronic reads only in the snRNA-seq data with GRCh38-2020-A library. For the human islet graft samples, which were also processed identically as the snRNA-seq dataset from in vitro islets, we included intronic reads as well and used the GRCh38-mm10-2020-A library to distinguish human and residual mouse genes. After the 10X h5 format file was generated, data were analyzed on the R platform with Seurat package V.4.1.1 [28].

### Quality control, integration, and projection

Ambient mRNA adjustment on the scRNA-seq, snRNA-seq, and in vivo snRNA-seq data was performed using SoupX (20% contamination estimation) [29]. Cells with less than a 500 gene count, less than 250 gene varieties, less than 0.8 log_10_ genes per UMI, and a greater than 20% mitochondrial gene ratio were filtered out. Then, doublets were algorithmically removed with the Doubltfinder package (20% estimation for in vitro scRNA/snRNA-seq, 10% estimation for in vivo snRNA-seq data) [30]. Datasets were projected to publicly available Azimuth’s Human Pancreas reference (https://azimuth.hubmapconsortium.org/references/human_pancreas/) according to the script template of demo data with resulting reduction and cell type annotation [7, 28, 31–36]. Finally, differential expression analysis within snRNA-seq datasets for identification of new gene sets was performed. The empirically based doublet finder hyperparameter following 10X genomics guidelines (https://kb.10xgenomics.com/hc/en-us/articles/360001378811-What-is-the-maximum-number-of-cells-that-can-be-profiled-) was an 8% doublet formation ratio per cell/nucleus recovered for the in vitro studies. Using this doublet formation rate, several quadruple hormonal clusters and scattered and un-clustered cells were observed when we used Azimuth-based reduction. However, when we used a 20% doublet rate [37], we found a cleaner edge, and a more defined UMAP structure than when using the empirically based hyperparameter.

Human in vivo islet datasets were projected onto our integrated scRNA/snRNA-seq dataset as a reference. Since human islet grafts from harvested mouse kidneys naturally contain residual mouse cells and their mRNAs, nuclei with more than 10% of mouse genes were filtered out during quality control process. For the in vivo data, we used an empirically based doublet finder hyperparameter as the 10X genomics table suggests and found that a 10% doublet rate is appropriate to define several human islet cell populations with cleaner cluster edges and more defined UMAP structures.

### Unsupervised data analysis

After data quality control, scRNA-seq and snRNA-seq data were integrated using Seurat’s SCTransform function without allocating method parameters [31]. Next, cell type identity was assigned according to the normalized gene expression level, referencing the canonical pancreatic cell type genes [28]. In vivo snRNA-seq data were created by integration among four samples of in vivo snRNA-seq data and cell types were annotated by referring to both canonical markers and newly found snRNA markers from this study.

### Pseudo-time and RNA velocity

After separating the β-cell cluster from the main data structure, we performed pseudo-time analysis using the monocle 3 package. First, we extracted gene and cell metadata from PCA feature and RNA counts. Monocle 3 uses a graph-based learning strategy by providing an inbuilt function to choose the base. It measures a vector distance between an interested cell along the path, and length is defined by the quantified transcriptional change along the trajectory inferred as RGE (reversed graph embedding). The learned-graph trajectory yielded one linear, non-branching transition overall. Subsequently, we recreated the monocle object with them, then UMAP coordinates were embedded into monocle’s reduction data to maintain the UMAP structure of the β-cell cluster. Using monocle 3’s built-in learn graph function by setting the “FALSE” hyperparameter for using partition, pseudo-time order was created by setting up the base at β3 cluster. The unbiased progression was also confirmed by the RNA velocity analysis [38, 39].

For RNA velocity analysis, we extracted cell identifier, PCA/UMAP coordinate, and metadata from main Seurat data object. Then we recreated matrix file with GetAssayData function from Seurat and writeMM from Matrix package. Loom file was created with Velocyto by referencing GRCh38 human genome for scRNA/snRNA-seq and GRCh38-mm10 for the human islet graft in vivo snRNA-seq. After loom file is obtained, cell identifiers (UMIs), UMAP coordinates, and β-cell subtype were embedded to AnnData with scVelo Package. From AnnData formatted data, spliced/unspliced counts were measured by proportions function of scVelo. With the stochastic modeling hyperparameter, we estimated the velocity and visualized with velocity streamline of the β-cell sub-clusters’ transitions [40, 41].

### Comparative analysis of β-cell subpopulations from previous published studies

To create the closest proximity to Dorrell et al. report data type [16], we created pseudo-bulk RNA sequencing data by taking average expression of the different β-cell subtypes in our study. Then, we visualized the expression of genes from Dorell et al. ([16], Fig. 4 in that report). To avoid the color-cue induced confusion, we adjusted our color scale with identical theme (blue- black-yellow) as well.

### Pathway analysis

To define the molecular and cell function, single-cell level gene set enrichment analysis was performed using the escape package which accesses the entire Molecular Signature Database (v.7.0) [30, 42–44]. The whole C2 library enrichment with chemical and genetic perturbations and canonical pathways containing five databases (Biocarta, KEGG, PID, Reactome, and Wikipathways) were employed. Additionally, the C5 (Gene Ontology) library was also investigated using keywords such as β-cell, pancreas, pancreatic, and exclusion keywords such as cancer, carcinoma, anomaly, or other pathologies. After enrichment scores were calculated for each single cell, they were added to the metadata for analysis and visualization.

### RNA in situ hybridization

RNA fluorescence in situ hybridization was performed on dispersed human islet cells using the RNA scope platform. Briefly, dissociated cells from human islets were plated on poly-D-lysine-coated coverslips and incubated for 30 min at 37 °C, 5%CO_2_. Cells were then fixed in 4% paraformaldehyde and in situ hybridization performed using the RNAscope® Multiplex Fluorescent Reagent Kit v2, probes Hs-ZNF385D (cat#1,161,581) targeting intron sequences in the region 808,843–810,453 of NC_000003.12:22,372,641–21,412,218 and Hs-ZNF385D (cat#116,501) targeting exon sequences in the region 150–1266 of NM_024697.3 which are present in *ZNF385D* pre-mRNA and mature mRNA respectively, and opal 620 (cat#FP1495001KT, Akoya Biosciences) following the instructions of the manufacturer (ACD Bio-Techne). Insulin immunolabeling was performed using anti-proinsulin/C-peptide antibody (cat# GN-ID4, DSHB), Alexa FluorTM 488 goat anti-rat IgG secondary antibody (cat#A11006, Invitrogen), and DAPI for nuclei detection.

### Statistical analysis

Data are presented as bar graphs, violin plots, and scatterplots and show means ± SE. Statistical significance analysis was performed using Wilcoxon rank-sum test or Student’s *t* test for comparison between groups as indicated in the figure legends. *P* < 0.05 was considered statistically significant. The simplified asterisk statistical significance annotation followed conventional criteria of 0.05, 0.01, 0.0001, and 0.0001 for increment number of asterisks.

### Study approval

All protocols were performed with the approval of and in accordance with guidelines established by the Icahn School of Medicine at Mount Sinai Institutional Animal Care and Use Committee (IACUC #2015–0107).

## Results

### RNA-seq profiling of cells and nuclei from adult human islets

**Fig. 1:**
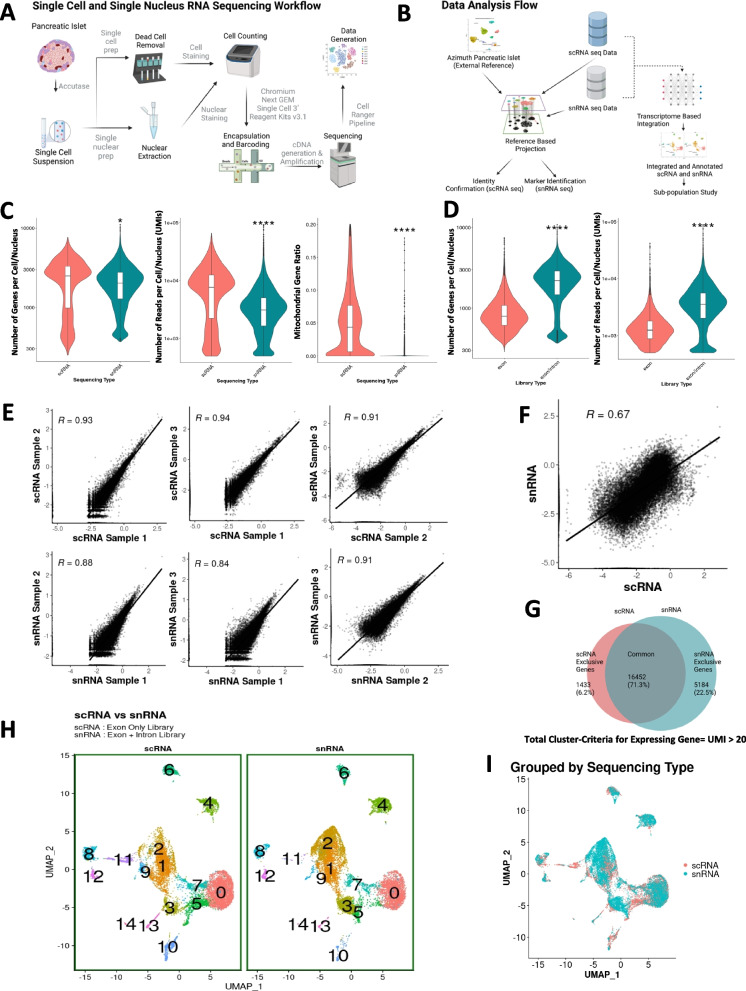
Experimental design, quality assessment, and unsupervised clustering. **A** Human islet processing and data generation scheme. **B** Data analysis workflow. **C** Number of genes per cell/nucleus, number of reads per cell/nucleus, and mitochondrial gene ratio comparison between scRNA-seq /snRNA-seq data. **D N**umber of genes and number of reads per cell/nucleus between snRNA-seq data with and without intronic reads. Statistical significance was tested using a Wilcoxon rank-sum test. **E** Average gene expression correlation among different human islet cell/nuclei preparations (*n* = 3 adult human islet donors). **F** Average gene expression correlation between RNA sequencing type (scRNA/snRNA). **G** Venn diagram of genes detected in both scRNA-seq and snRNA-seq analysis of the three human islet samples, UMI > 20. **H** Unsupervised clustering of scRNA-seq and snRNA-seq integrated data with Louvain resolution of 0.8**. I**. Dimensional reduction plot grouped by RNA sequencing type

Figure 1A and B depict the approach and data analysis workflow used for these studies. Islets from three healthy adult human islet donors (Additional file 1: Table S1) were used. Islet cells and nuclei from each donor were analyzed side-by-side. Cells from 3000 IEQs from each of the human islet preparations were dispersed, nuclei extracted from half of the cells and the other half went through the dead cell removal process kit. After quality assessment and counting, 5000–16,000 cells or nuclei for each sample were loaded into the 10X Genomics Chromium Controller, poly-A transcripts reversed transcribed and amplified, cDNA tagmented, and the resulting libraries sequenced to a depth of 250–500 million reads per sample (Additional file 1: Table S2). scRNA-seq and snRNA-seq data were projected onto the Azimuth human pancreas reference to determine islet cell populations and identify new gene sets as markers for these cells. In addition, scRNA-seq and snRNA-seq data were first integrated and then separated for the analysis of different β-cell subpopulations.

Outlier cells potentially representing low-quality cells or multiple cell captures were removed by excluding those with very low or high UMIs. Therefore, cells with either log-normalized UMI/cell-nucleus or gene/cell-nucleus values > 2.5 standard deviations (SDs) or <  − 2.5 SDs from the median value were removed [45]. Cells and nuclei with high expression of mitochondrial genes with a cutoff of 20% were also removed. Estimated ambient RNA contamination was set up to 20% for the SoupX algorithm, and data were adjusted accordingly. RNA contamination in the scRNA-seq and snRNA-seq dataset were similar (contamination estimator rho ranged between 0.02 and 0.07). Ultimately, 10,732 cells (4395, 4137, and 2200 cells per human islet preparation) and 11,018 nuclei (3033, 6533, and 1452 nuclei per human islet preparation) were analyzed. The number of genes and reads sequenced per cell was lower in the snRNA-seq compared to the scRNA-seq approach (Fig. 1C) but the ratio between usable versus sequenced reads determining sequencing efficiency was similar in both methods (0.974 ± 0.002 scRNA-seq vs. 0.966 ± 0.002 snRNA-seq). The percentage of mitochondrial genes sequenced in the nuclei preparations were below 1% and clearly and significantly lower than the mitochondrial genes sequenced in the cell preparations (Fig. 1C). As expected, the number of genes and reads was significantly higher when intron plus exon reads were analyzed compared with exon reads alone in snRNA-seq data (Fig. 1D).

Based on the data quality above, we next compared scRNA-seq using exon reads with snRNA-seq using both intron and exon reads for improved gene detection and mapping as previously done [23, 46]. scRNA-seq analyzes both nuclear and cytoplasmic transcripts with a majority being cytoplasmic, whereas snRNA-seq profiles mostly nuclear transcripts with minimal transcripts derived from cytoplasm or rough ER during nuclei isolation [47, 48]. Therefore, we expected that RNA-seq reads would be different in the scRNA and snRNA sequencing profiles. In cells, 17 ± 0.7% reads were intronic reads, while in nuclei these were 53 ± 1.9%. On the other hand, in cells 73 ± 1.6% reads were exonic reads in contrast to 32 ± 0.8% in nuclei. As expected, therefore, complete or near complete linearity of gene expression correlation occurred only when nuclei were compared to nuclei or cells to cells (*R* = 0.91–0.93) (Fig. 1E), while this correlation was lower when cells were compared with nuclei (*R* = 0.67) (Fig. 1F). Indeed, the number of common genes detected by both RNA sequencing approaches (UMI > 20) was 16,452 (71.3%), while 1433 genes (6.2%) were exclusively detected in scRNA-seq, and 5184 genes (22.5%) were exclusively detected in snRNA-seq (Fig. 1G). This indicates that almost 29% of the genes detected by both approaches are different for the same human islet samples suggesting that the two RNA sequencing methods might reveal differences in the identity of islet cell populations. Even using a range of UMIs from 0.1 to 100 to determine the number of common genes detected, differences in gene detection were 13, 15, 27, 29, 30, and 32% (Additional file 1: Fig. S1). Interestingly, unsupervised clustering of cells and nuclei using exonic reads (scRNA-seq) or intronic plus exonic reads (snRNA-seq) revealed clusters with similar locations and with strong overlap in their UMAPs using Seurat’s integration algorithm (Fig. 1H,I) [49].

### Supervised classification of human islet cell types with scRNA-seq and snRNA-seq

**Fig. 2:**
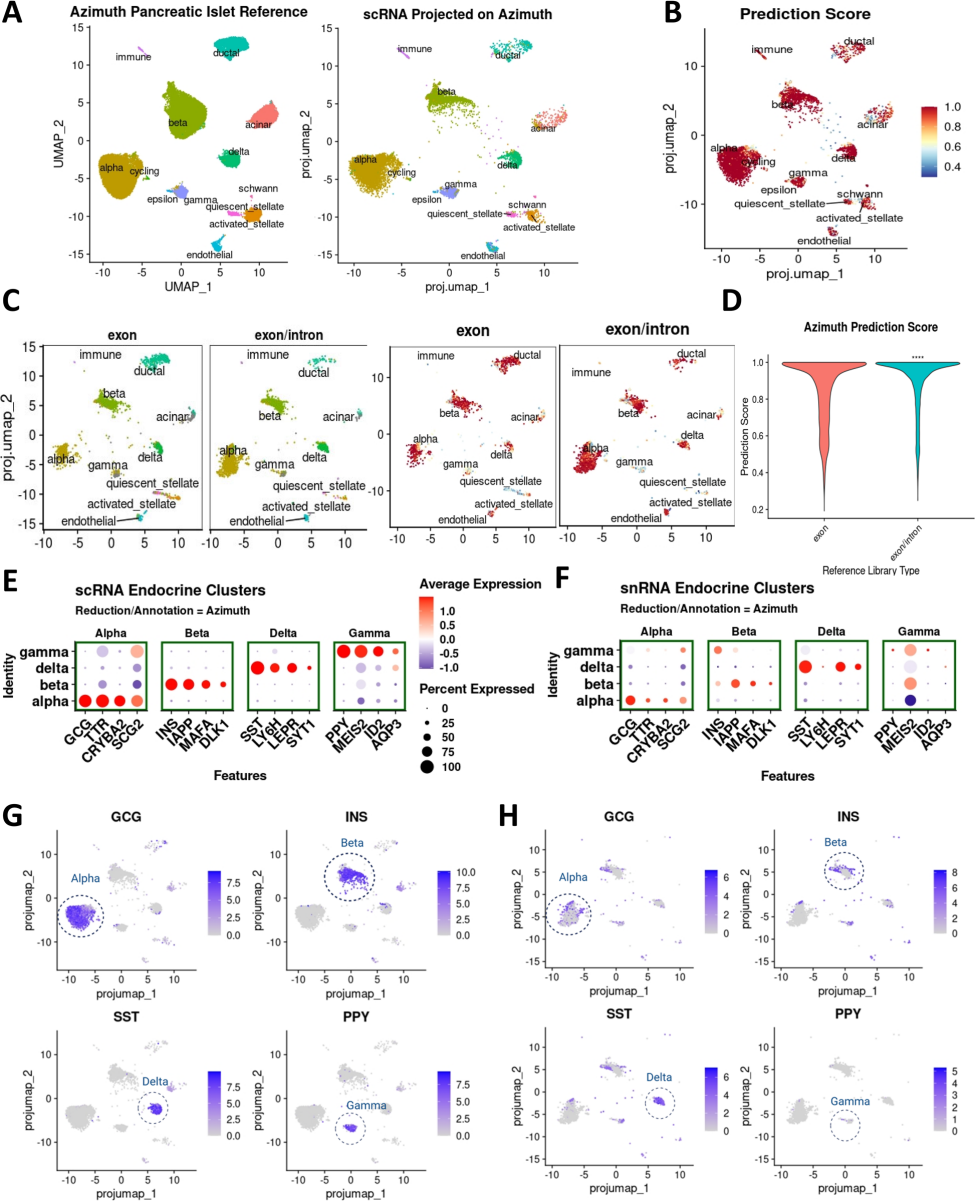
Human islet cell type identification by projection strategy and mapping score assessment. **A** Azimuth pancreatic islet reference v1.0.1 (left) and scRNA-seq data projected on the Azimuth reference (right). **B** Cell type annotation prediction score displayed on dimensional reduction plot. **C** snRNA-seq data with only exon reads or with both exon and intron reads projected on the Azimuth reference (left), cell type projection score on dimensional reduction plot (right). **D** Prediction score in the snRNA-seq analysis with only exon reads or with both exon and intron reads in the projections in **C**. Statistical analysis using a Wilcoxon rank-sum test indicates a significant higher prediction score for the snRNA-seq data projection using exon plus intron reads. **E** Expression level and percentage of cells expressing canonical genes of Azimuth annotated endocrine cell types in the scRNA-seq data. **F** Expression level and percentage of cells expressing canonical genes of Azimuth annotated endocrine cell types in the snRNA-seq data. **G** Dimensional reduction plot for four representative canonical endocrine genes in the scRNA-seq data. **H** Dimensional reduction plot for four representative canonical endocrine genes in the snRNA-seq data

We next projected the scRNA-seq and snRNA-seq datasets onto a publicly available Azimuth integrated human pancreas reference that comprises six different scRNA-seq datasets generated using several different single-cell technologies employing Seurat (Figs. 1B and 2A–D) [7, 28, 31–36]. Projection was done with exonic reads for scRNA-seq (Fig. 2A,B) and exonic or intronic plus exonic reads for snRNA-seq (Fig. 2C,D). When we projected the human islet scRNA-seq data of the current study onto the reference, we found that there was a near perfect alignment of the different human islet cell populations with a prediction score median of 1, and a mean of 0.948 (Fig. 2A,B). Projection of the snRNA-seq data to the reference using exonic reads or intronic plus exonic reads also led to a high degree of alignment (Fig. 2C,D) with a prediction score significantly higher with exon plus introns than with exons alone (median 0.967 vs 0.944, and mean 0.898 vs 0.861) (Fig. 2D). To determine whether the observed higher probability of alignment to a reference with exon plus intron reads could be explained by different UMI depth, we subsampled the intron-inclusive library with the median value of 1247 UMIs (median of exonic reads only library) and analyzed the reference alignment prediction score. We found that the alignment prediction score was still superior with the intron-inclusive library than with the exon only library following subsampling (Additional file 1: Fig. S2). This further validates the use of information from intron plus exon reads for detailed analysis of human islet cell populations using snRNA-seq. The data also indicate that human islet cell clusters from scRNA-seq and snRNA-seq share a high degree of similarity and that snRNA-seq data containing intron plus exon reads are interchangeable with scRNA-seq data for the identification of human islet cell type clusters.

Next, we tested the association of the different clusters generated from the scRNA-seq and snRNA-seq data (Fig. 2A, C) with gene expression levels of canonical genes in different endocrine cell clusters (Fig. 2E–H). A strong correlation was observed between established canonical gene cell markers (*GCG*, *INS*, *SST*, and *PPY*) along with several other known selective markers with α-, β-, δ-, and γ-cells in scRNA-seq. Thus, cell types in the UMAP were accordingly assigned in the scRNA-seq analysis (Fig. 2E, G, Additional file 2: Data S1). However, the strong correlation of these canonical gene markers observed in scRNA-seq was weaker in the snRNA-seq for α-, β-, and γ-cells (Fig. 2F, H, Additional file 3: Data S2). For example, in β-cells, *INS* and *IAPP* are the top differentially expressed genes, whereas they are not the top differentially expressed genes in the snRNA-seq datasets. Furthermore, even after subsampling the UMI to median of snRNA-seq (which is lower than scRNA-seq, Fig. 1C), the pattern of expression level of canonical genes is weaker in the snRNA-seq for α-, β-, and γ-cells (Additional file 1: Fig. S2B). These results highlight the need for the identification of new gene sets as markers for islet endocrine cells that more appropriately define them in the snRNA-seq analysis.

### Novel gene sets in the snRNA-seq dataset for the identification of human islet endocrine cell types

**Fig. 3:**
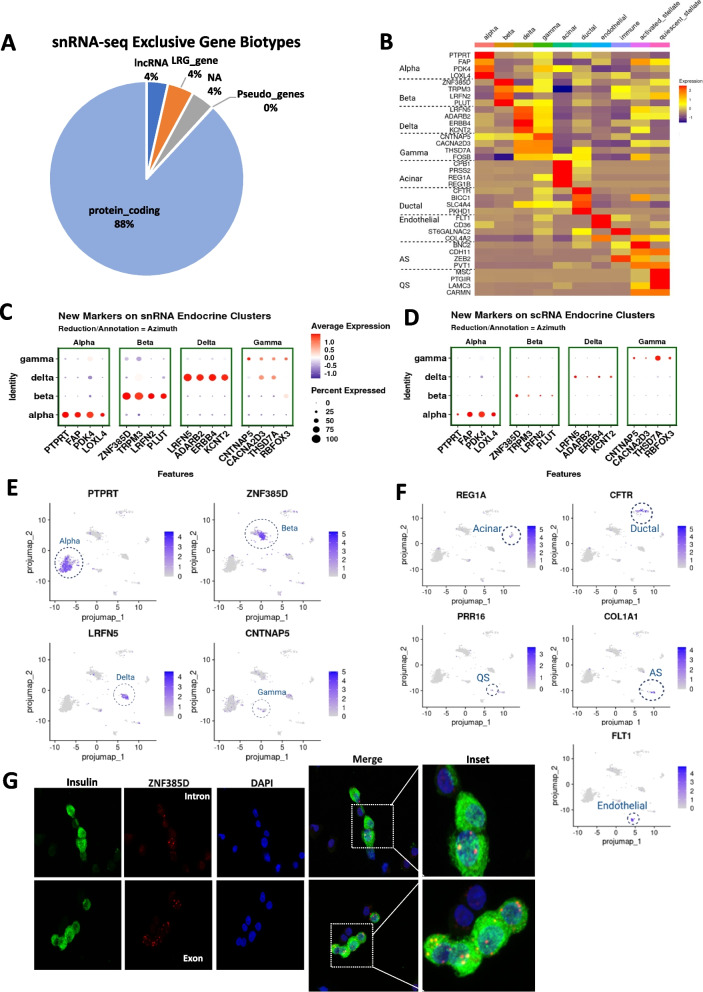
Identification of unique snRNA-seq gene markers in human islet endocrine cells. **A** snRNA-seq identification of gene biotypes referring to AnnotationHub [50]. **B** Identification of differentially expressed genes with a *p* value ~ 0 and log2-Fold change greater than 1.5 for each cell cluster and log2FC < 0.85 in the snRNA-seq data using pseudo-bulk averaged heatmap. Four top genes are presented. **C** Dotplot projection of newly found gene markers from the snRNA-seq data on Azimuth annotated endocrine clusters. **D** Dotplot projection of newly found gene markers in the snRNA-seq dataset on Azimuth annotated endocrine clusters for scRNA-seq data. **E** Projection of the four top newly found gene makers for endocrine cells from the snRNA-seq data on UMAP: *PTPRT* (α-cells), *ZNF385D* (β-cells), *LRFN5* (δ-cells), and *CNTNAP5* (γ-cells).** F** Projection of the four top gene makers for exocrine cells from the snRNA-seq data on UMAP: *REG1A* (acinar cells), *CFTR* (ductal cells), *FLT1* (endothelial cells), *COL1A1* (activated stellate cells), *PRR16* (quiescent stellate cells). **G** Representative images of RNA fluorescence in situ hybridization performed in dispersed human islet cells using the RNA scope platform with probes targeting *ZNF385D* (green) intron sequences present only in pre-mRNA (top) and exon sequences present in both mature mRNA and pre-mRNA. Insulin immunofluorescence (green) was used to detect β-cells and DAPI for nuclei detection (blue)

We next performed differential gene expression analysis between scRNA-seq and snRNA-seq samples and investigated the biotype of the snRNA-seq enriched genes. Even with the inclusion of intronic reads, most of the genes in snRNA-seq are protein-coding genes (Fig. 3A). Next, we annotated our snRNA-seq data with projection onto Azimuth’s human pancreas reference and tested differential gene expression for each cluster with the entire dataset (Fig. 1B). Using this approach, differentially expressed genes in each cell cluster were identified with a *p* value ~ 0 and log_2_FC greater than 1.5. Even if the candidate genes were qualified by these criteria, we omitted genes that showed considerable expression (log_2_FC > 0.85) in other cell clusters. Thereby, we compiled a list with the top four differentially expressed genes for each cell type (Fig. 3B and Additional file 3: Data S2). Interestingly, none of the top four differentially expressed genes in scRNA-seq that define endocrine cells, i.e., *INS*, *GCG*, *STS*, or *PPY* appear in this short list of differentially and selectively expressed genes in snRNA-seq. This indicates that these canonical genes are not the top differentially expressed genes in the snRNA dataset among α-, β-, δ-, and γ-cells. To confirm the reliability of these newly identified gene markers in endocrine cells, we tested them on the snRNA-seq and scRNA-seq data objects. They showed a clear and mostly exclusive pattern of expression in the corresponding cell clusters both in the snRNA-seq and scRNA-seq, but with higher expression in the snRNA-seq datasets (Fig. 3C,D). In particular, top endocrine cell gene markers (*PTPRT*, *ZNF385D*, *LRFN5*, and* CNTNAP5* for α-, β-, δ-, and γ-cells, respectively) displayed a more distinctive localization pattern than their corresponding canonical single-cell clustering gene markers (*GCG*, *INS*, *SST*, and *PPY*) for α-, β-, δ-, and γ-cells, respectively, in the snRNA-seq data objects (Figs. 2H and 3E). Of note, *CNTNAP5* did not display highly different expression pattern in γ-cells, yet it was considered a γ-cell gene marker based on high adjusted *p* value (1.58 × 10^−5^) and log_2_FC of 1.654. Interestingly, the top differentially expressed genes in the snRNA-seq analysis in non-endocrine cells contained the canonical gene markers that define these cell types in scRNA-seq (*REG1A*, *CFTR*, *FLT1*, *COL1A1*, and *PRKG1* for acinar, ductal, endothelial, activated stellate, quiescent stellate cell, respectively). This suggests an interesting dichotomy between human endocrine and non-endocrine cells regarding steady-state transcript abundance of canonical genes (Fig. 3F). To gain confidence on the usefulness of the new gene sets for snRNA-seq analysis of human islet cells, we analyzed the only previously published snRNA-seq dataset of isolated human islets [22] but using exon + intron reads. We projected the snRNA-seq data into the Azimuth integrated human pancreas reference and analyzed the expression levels of the novel gene sets. As shown in Additional file 1: Figs. S3A and S3C, the novel gene sets described above showed a clear and mostly exclusive pattern of expression in the corresponding cell cluster; however, the number of nuclei in this study was limited, 471 nuclei, and no alpha cells were detected.

To validate the presence of these newly identified genes from the snRNA-seq analysis as cell markers, we focused on β-cells and performed RNA scope to detect *ZNF385D* mRNA in dispersed human islet cells from healthy donors (Additional file 1: Table S1). As shown in Fig. 3G, *ZNF385D* mRNA expression was clearly and uniquely detected in human β-cells. Expression using an intronic probe was limited to the nucleus (Fig. 3G, top) while using an exonic probe located the signal in both the cytoplasm and the nucleus (Fig. 3G, bottom).

### Comparative scRNA-seq and snRNA-seq analysis distinguishes three different β-cell subtypes

**Fig. 4:**
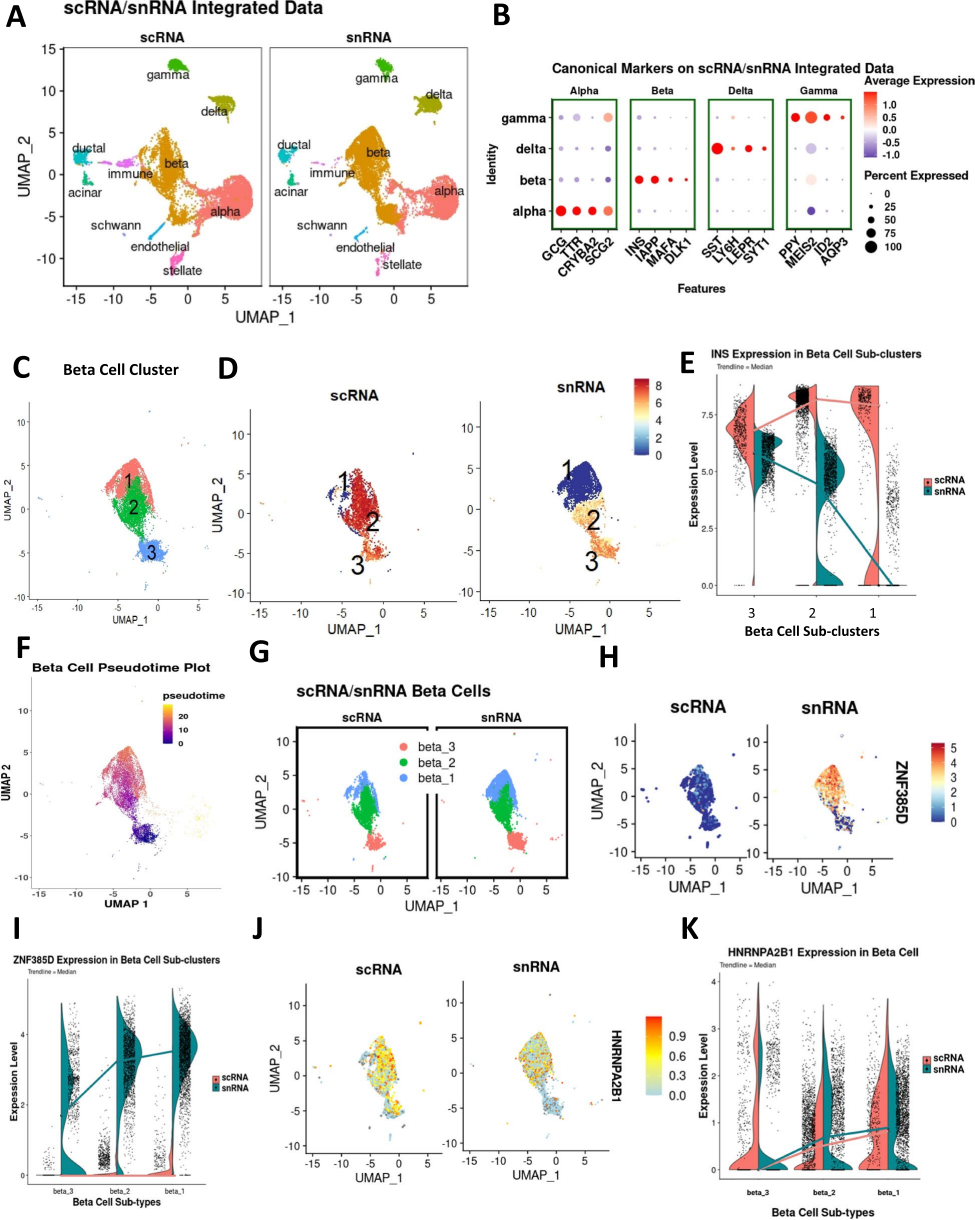
Human β-cell subtypes using unsupervised scRNA-seq and snRNA-seq data integration. **A** Integrated and self-annotated clusters on UMAPs of scRNA-seq and snRNA-seq data. **B** Canonical endocrine cell gene marker expression on scRNA-seq and snRNA-seq integrated dataset. **C** Separated β-cell cluster from integrated main data identifying three cell sub-clusters using a Louvain resolution of 0.4. **D** *INS* expression pattern in the β-cell cluster in scRNA-seq and snRNA-seq data on UMAP. **E** *INS* expression level on violin plots in the three β-cell subtypes in the scRNA-seq and snRNA-seq data. **F** Monocle 3 generated pseudo-time dimensional reduction plot in the separated β-cell cluster from integrated main data using lower *INS* expression area in scRNA-seq data (cluster 3) as base. **G** Re-annotated β-cell sub-clusters of scRNA-seq and snRNA-seq data based on pseudo-time *INS* expression. **H** *ZNF385D* expression pattern in the β-cell sub-clusters in scRNA-seq and snRNA-seq data on UMAP. **I** *ZNF385D* expression level on violin plots in the three β-cell subtypes in the scRNA-seq and snRNA-seq data. **J** *HNRNPA2B1* expression pattern in the β-cell sub-clusters in scRNA-seq and snRNA-seq data on UMAP. **K** *HNRNPA2B1* expression level on violin plots in the three β-cell subtypes in the scRNA-seq and snRNA-seq data

To identify human β-cell subtypes within the β-cell cluster, we created a new data object integrating scRNA-seq and snRNA-seq datasets to compare *INS* gene expression patterns (Figs. 1B and 4A–C). Combining the two datasets effectively increased the power of the analysis while providing additional information from both cytoplasmic and nuclear transcriptomes. After sub-setting the identified β-cell cluster, we generated clusters with a Louvain resolution of 0.8 (Fig. 4C) to assign three β-cell sub-clusters. *INS* gene expression in the β-cell clusters between scRNA- and snRNA-seq data objects was different in terms of topographical location (Fig. 4D,E and Additional file 4: Data S3). Cluster 3 displayed lower *INS* expression in scRNA-seq data but the highest in snRNA-seq data object (Fig. 4E), suggesting that cluster 3 includes β-cells with mainly *INS* pre-mRNA since snRNA-seq analyzes mostly pre-mRNA. Similar results were observed when a previous snRNA-seq dataset from human islets [22] was analyzed (Additional file 1: Figs. S3D and S3E).

Detailed analysis of cells in between clusters 2 and 3 following cluster separation with a high Louvain algorithm resolution of 2.0 to resolve smaller clusters (Additional file 1: Fig. S4A) identified cluster 23 as the cluster in between clusters 2 and 3 (Additional file 5: Data S4). Differential gene expression analysis of cluster 23 (transitioning) cluster against all remaining clusters showed high expression levels of mostly ribosomal protein genes (Additional file 1: Fig. S4B-C). GSEA (Reactome) depicted biological translation elongation and termination as the top biological processes in these inter-cluster 2–3 cells (Additional file 1: Fig. S4D).

Next, we created pseudo-time and RNA velocity trajectory graphs, assigned cluster 3 as the base (Fig. 4F, and Additional file 1: Fig. S5) and rearranged the order of each cluster according to the pseudo-time or RNA velocity trajectory into β1, β2, and β3 cells (Fig. 4G and Additional file 1: Fig. S5A-B). Similar progression from cluster 3 to cluster 1 occurred by using integrated data from both datasets in pseudo-time or RNA velocity analysis. RNA velocity analysis of individual scRNA-seq data showed similar transition from cluster 3 to cluster 1. Interestingly, RNA velocity analysis of only snRNA-seq data showed bifurcation from the end of cluster 3 to edge of cluster 3 and then from cluster 2 to cluster 1 (Additional file 1: Fig. S5B). These results indicate that identification and transition among the β-cell sub-clusters can be observed with integrated or individual RNA-seq data but this transition seems to differ between scRNA-seq and snRNA-seq for cluster 3. This can very likely be because RNA velocity is based on the ratio of exon-to-intron reads, leveraging the fact that newly transcribed, unspliced mRNAs infer a time derivate of gene expression state, but exon-to-intron reads are low in snRNA-seq (Additional file 1: Fig. S5A) complicating the interpretation of the transition among clusters. Therefore, for integrated or scRNA-seq data, RNA velocity is appropriate but for snRNA-seq it seems that RNA velocity is less optimal for analyzing the progression of β-cell maturation.

Since cells in the β1 cell cluster have stable *INS* expression as inferred from the scRNA-seq (mature mRNA) β-cell cluster object, but very low *INS* expression as inferred from the snRNA-seq (pre-mRNA) β-cell cluster object, we considered these cells as the *INS*-rich cell subpopulation. Next, we looked at *ZNF385D* expression, the highest differentially expressed gene in snRNA-seq in β-cells and found minimal expression in the β-cell sub-clusters in the scRNA-seq data object but different topographical location in the snRNA-seq data object (Fig. 4H,I) where β1 represents cells with high *ZNF385D* pre-mRNA expression, opposite to *INS* expression (Fig. 4E). Next, we looked at the expression of the *INS* mRNA-binding protein *HNRNPA2B1*, an RNA-binding protein that regulates *INS* mRNA stability and translation [51], to determine whether the β2 cell cluster represents a transition stage between β3 and β1. As shown in Fig. 4J,K, *HNRNPA2B1* expression is increased in clusters 2 and 1 compared to cluster 3, suggesting that cluster 2 may represent an “in transition” β-cell type in which cells go from transcriptionally active β3 cells to β1 cells with mature stored *INS* mRNA.

Furthermore, we also performed a comparison analysis of β-cell subtypes described in Dorrell et al. [16] with β-cell subpopulations in our study. We referred gene sets from the RNA-seq data in Dorell et al. study ([16], Fig. 4 in that report) and visualized gene expression by average heatmap (Additional file 1: Fig. S6A-B). Gene sets for ST8SIA1^−^ β1/β2 (*HCN4* to *G6PC2*) and ST8SIA1^+^ β3/β4 (*KCNE4* to *SIX3*) in the Dorrell et al. report had a mixed pattern in our datasets (Additional file 1: Fig. S6A). However, we find that gene sets for CD9^−^ β1/β3 (*FKBP5* to *NPY*) and CD9^+^ β2/β4 (*CD9* to *EPB41L1*) in the Dorrell et al. report had a closer correlation with our dataset (Additional file 1: Fig. S6B). Indeed, gene set expression in β3 correlated with Dorrell et al. β2/β4 and the current β1 correlated with Dorrell et al. β1/β3. Expression of *CD9* corresponded well with their study. In addition, β1/β3 in the Dorrell et al. report showed the highest GSIS response and the current β1 cells showed the highest expression level of genes involved in the regulation of insulin secretion pathway (see below). Moreover, the current β2 cells showed a transitional expression pattern between β3 and β1.

### Gene pathway analysis in the three different β-cell subtypes

**Fig. 5:**
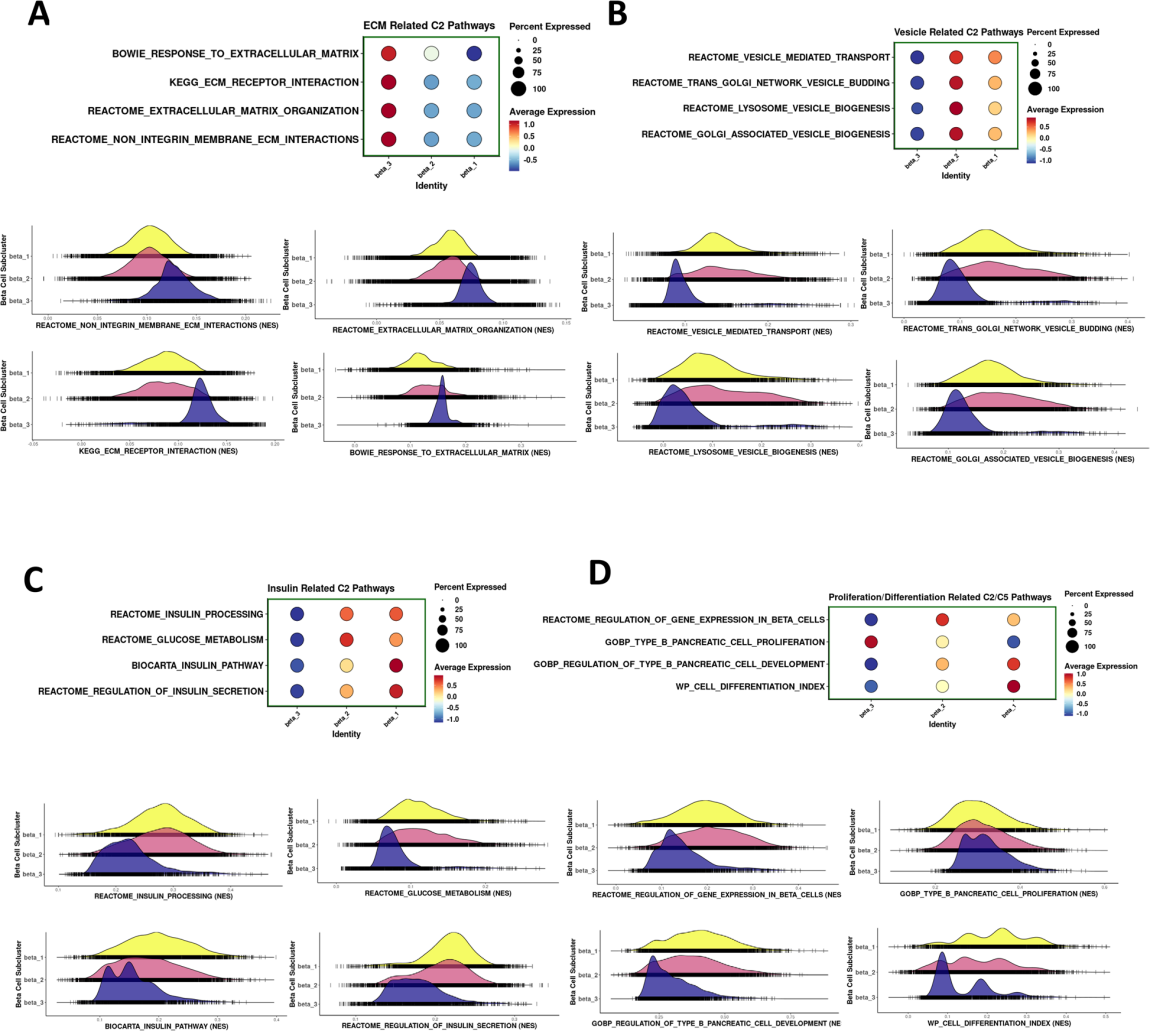
Cellular processes identified by gene set enrichment analysis (GSEA) of the three β-cell sub-clusters β1, β2, and β3 in the scRNA-seq and snRNA-seq integration dataset. **A** Dotplot depicting the expression levels and percentage of cells expressing four representative extracellular matrix (ECM)-related C2 pathway genes (interaction, response, organization, and integrins) and the corresponding enrichment plots below. **B** Dotplot depicting the expression levels and percentage of cells expressing four representative vesicle-related C2 pathway genes (biogenesis, budding, transport, lysosome) and the corresponding enrichment plots below. **C** Dotplot depicting the expression levels and percentage of cells expressing four representative insulin-related C2 pathway genes (processing, secretion, and glucose metabolism) and the corresponding enrichment plots below. **D** Dotplot depicting the expression levels and percentage of cells expressing four representative proliferation/differentiation related C2 pathway genes (proliferation, development, differentiation, regulation of gene expression) and the corresponding enrichment plots below

To research the potential biological differences among β3, β2, and β1, we performed GSEA of the combined datasets from scRNA- and snRNA-seq experiments [42–45]. Interestingly, enrichment of genes that define the biological processes of extracellular matrix (ECM) formation, interaction, and response were uniquely present in the β3 sub-cluster (Fig. 5A). Equally, GSEA also indicated that the biological processes of intracellular vesicle budding, transport, and formation are mainly present in the β2 sub-cluster (Fig. 5B), while genes for the biological processes of insulin secretion, processing, and glucose metabolism are mainly represented in the β2 and β1 sub-clusters (Fig. 5C). Additional information was also obtained on several biological processes of importance for the development, differentiation, gene expression regulation, and proliferation of the β-cell when we input the datasets from both RNA-seq approaches into the GSEA. As shown in Fig. 5D, the genes involved in β-cell proliferation were more highly expressed in the β3 sub-cluster, the genes involved in the regulation of gene expression were prominent in the β2 sub-cluster while the genes involved in β-cell development and differentiation were more highly expressed in the β1 sub-cluster. These results clearly delineate different sets of genes for specific cell functions in the different β-cell sub-clusters emphasizing the heterogeneity of β-cells in the human islet.

### Single-nucleus RNA-seq analysis of human islets in vivo

**Fig. 6:**
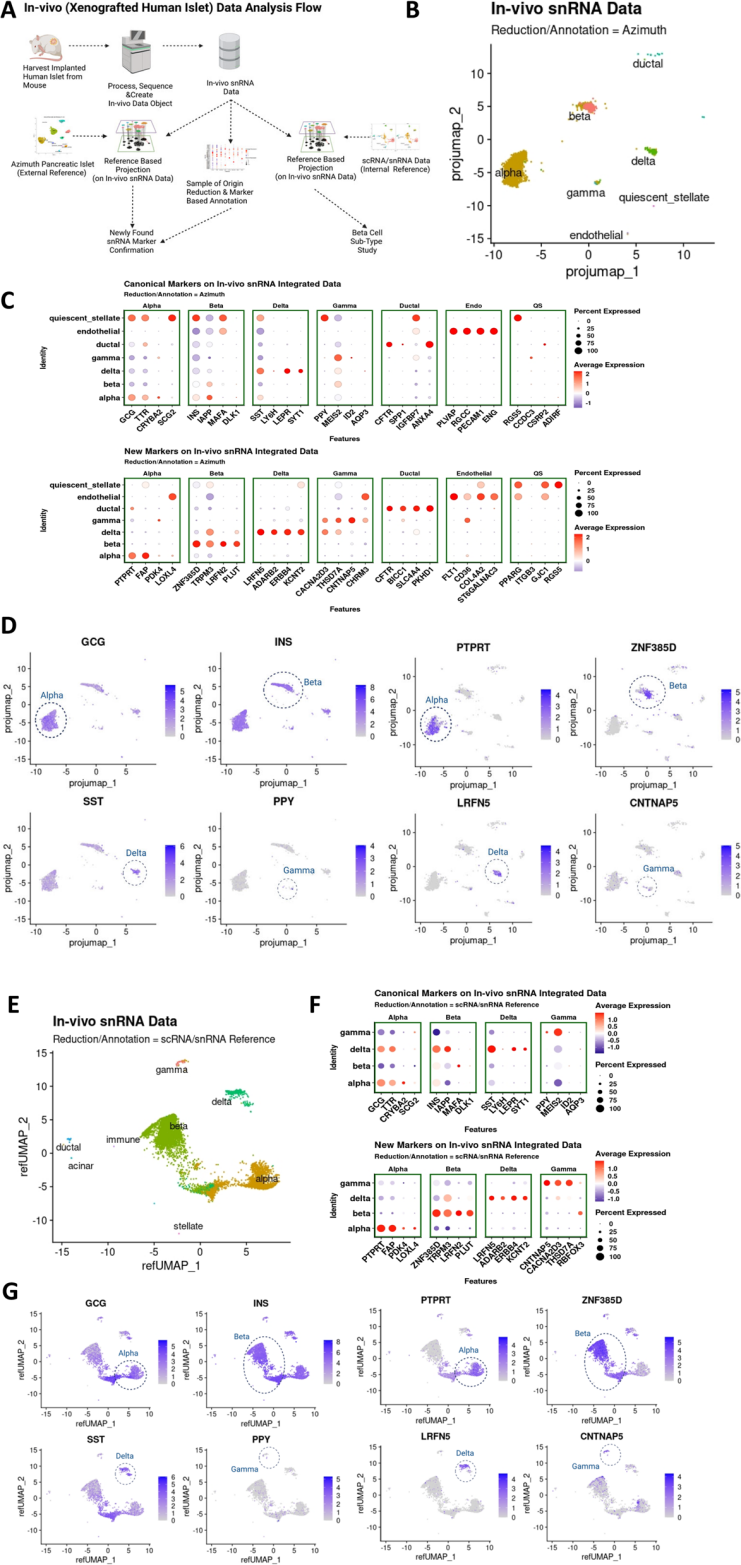
Single-nucleus RNA-seq analysis of human islets in vivo in xenografts in immunosuppressed mice. **A** Human islet grafts processing and data analysis scheme. **B** In vivo snRNA-seq data obtained from four different human islet xenografts done with four different human islet preparations from adult human islets from healthy donors projected on Azimuth pancreatic islet reference and cluster annotation. **C** Dotplot depicting gene expression and percentage of cells expressing canonical gene markers (top) and newly identified gene markers for endocrine cells on Azimuth annotated in vivo data. **D** Projection of canonical genes and the four top newly found gene makers for endocrine cells from the in vivo snRNA-seq data on the dimensional reduction plot by Azimuth. **E** In vivo snRNA-seq data projected on internal reference with scRNA-seq and snRNA-seq of human islets in vitro integrated data and annotated. **F** Dotplot depicting the expression levels and percentage of cells expressing the canonical gene markers (top) and newly identified markers in the snRNA-seq of human islets in vitro (bottom) on internal reference annotated in vivo snRNA-seq data. **G** Projection of canonical genes and the four top gene makers for endocrine cells found in the snRNA-seq data from in vitro human islets of the in vivo snRNA-seq data on the dimensional reduction plot by internal reference

Next, we sought to examine the different human islet cell populations in vivo using human islet grafts transplanted in euglycemic immunosuppressed mice. For this purpose, we transplanted 1000 human IEQs from four healthy donors into the kidney capsule of RAG1^*−*/*−*^ mice and harvested the graft 3 months after transplantation (Fig. 6A). Nuclei were directly extracted from the grafts by the Minute™ single nucleus isolation kit and 5000–16,000 nuclei per sample after quality assessment and counting were loaded into the 10X Genomics Chromium Controller, poly-A transcripts reversed transcribed and amplified, cDNA tagmented, and the resulting library sequenced to a depth of 250–500 million reads per sample (Additional file 1: Table S2). Ultimately, 7765 nuclei (1632, 1509, 3911, and 713 nuclei per human islet preparation) were analyzed. The number of genes was 2226 ± 263, the number of gene counts was 4125.64 ± 263, and the ratio between usable versus sequenced reads determining sequencing efficiency was 0.999 ± 0.001. The percentage of mitochondrial genes sequenced in the nuclei preparations were below 1%. snRNA-seq data were projected onto the Azimuth human pancreas reference to identify islet cell populations using canonical gene sets as well as the new gene sets described earlier (Figs. 1A and 6A). In addition, the snRNA-seq data were projected onto the integrated scRNA-seq and snRNA-seq data from the in vitro studies for the analysis of different β-cell subpopulations (Fig. 6A). Gene counts and/or UMI count outliers or cells with high expression of mitochondrial genes or with ambient RNA contamination ≥ 20% were removed. During the quality control process, we found that ≤ 10% mouse gene ratio was optimal for identifying different cell types in vivo in this islet transplant setting without considerable mouse gene influence on the clustering pattern.

Using the reference-based reduction and annotation with Azimuth, we confirmed seven distinct human islet cell clusters, primarily comprised of endocrine cells (Fig. 6B). For simplicity, we omitted clusters with less than five cells from the dataset. The set of gene markers identified by the snRNA-seq analysis in vitro (Fig. 3C) showed clearer patterns of specific cell expression in both the average expression-based dotplot (Fig. 6C and Additional file 6: Data S5) and the scatterplot (Fig. 6D) compared to the non-specific pattern of the canonical gene markers. This specific cell cluster alignment of the newly identified gene sets from the snRNA-seq analysis in vitro persisted when an internal reference-based reduction and annotation (the scRNA-seq plus snRNA-seq integrated datasets from the in vitro samples, unsupervised, and marker annotated) was used (Fig. 6E–G). To gain confidence on the usefulness of the new gene sets for snRNA-seq analysis of human islet cells in grafts, we analyzed the previously published snRNA-seq dataset of human islet grafts by Basile et al. [22] using exon + intron reads. As shown in Additional file 1: Fig. S3B-C, the novel gene sets showed a clear and mostly exclusive pattern of expression in the corresponding cell clusters, validating the new gene sets as specific markers of cell types.

### β-cell subtypes in human islets in vivo

**Fig. 7:**
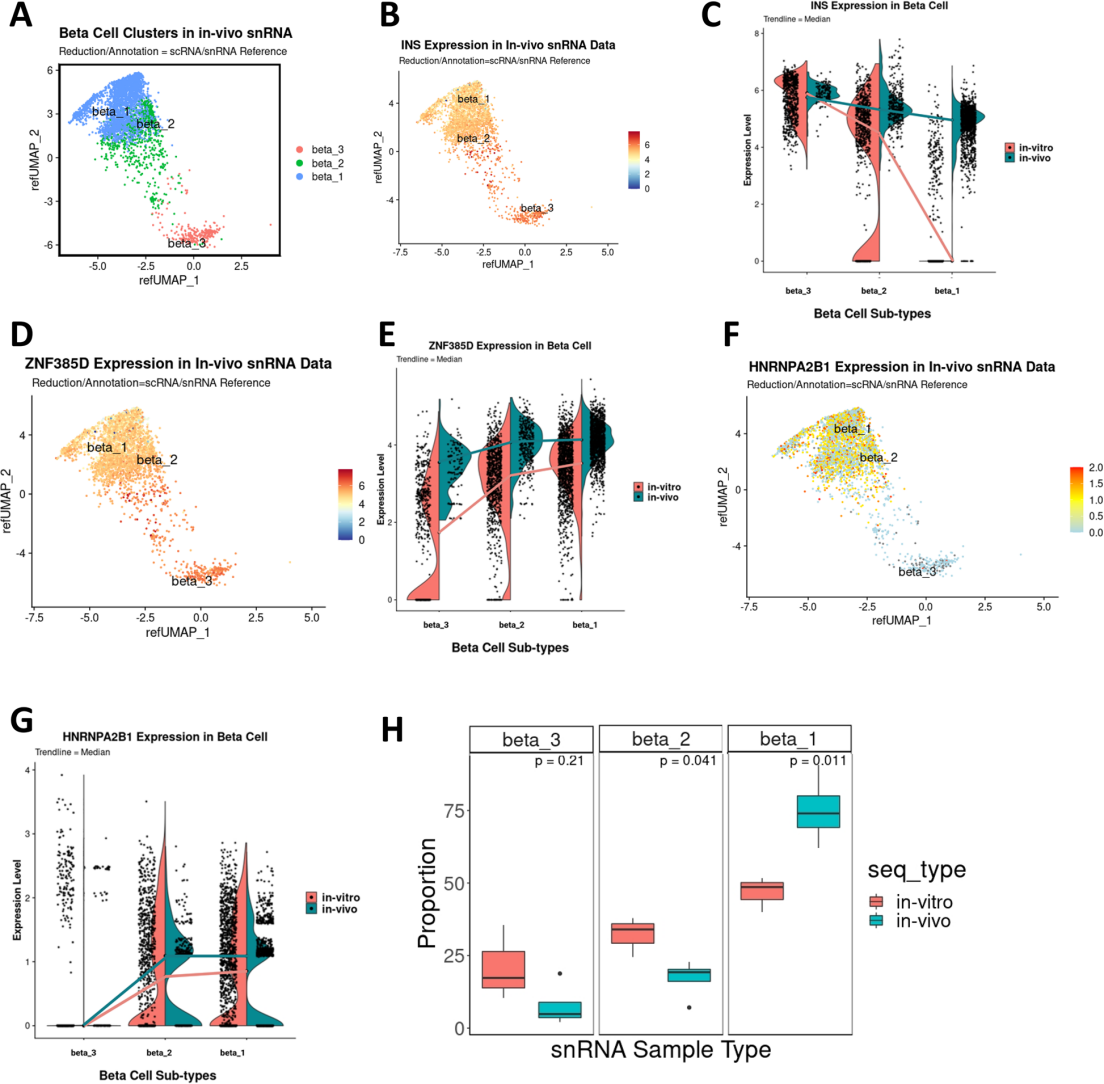
Human β-cell subtypes in vivo from the snRNA-seq data by projection on the in vitro internal reference. **A** Separated β-cell cluster from integrated main data identifying three cell sub-clusters using a Louvain resolution of 0.4. **B** *INS* expression pattern in the β-cell cluster in the in vivo snRNA-seq data on UMAP. **C** *INS* expression level on violin plots in the three β-cell subtypes in the in vivo snRNA-seq data compared with the in vitro snRNA-seq data. Notice the difference in expression in the β1 sub-cluster. **D** *ZNF385D* expression pattern in the β-cell sub-clusters in the in vivo snRNA-seq data on UMAP. **E** *ZNF385D* expression level on violin plots in the three β-cell subtypes in the in vivo snRNA-seq data compared with the in vitro snRNA-seq data. **F** *HNRNPA2B1* expression pattern in the β-cell sub-clusters in the in vivo snRNA-seq data on UMAP. **G** *HNRNPA2B1* expression level on violin plots in the three β-cell subtypes in the in vivo snRNA-seq data compared with the in vitro snRNA-seq data. **H** Proportion of the different β-cell subtypes in vivo and in vitro. Notice that in vivo, β1 cells become the majority of β-cells. Statistical analysis by Student’s *t* test indicates a significant increase in vivo of β1 cells while β2 and β3 cell subtypes are reduced

First, we determined whether the β-cell subtypes identified in the scRNA-seq/snRNA-seq in vitro study were still present in human islets in the in vivo setting. Thus, we projected the in vivo snRNA-seq dataset onto the in vitro scRNA-/snRNA-seq dataset reference which is pre-labeled with β-cell subtypes (Fig. 6E) and extracted the β-cell cluster (Fig. 7A and Additional file 4: Data S3). We separated β-cell sub-clusters from the main data cluster and examined the gene expression patterns (Fig. 7A–H). Interestingly, and in contrast to the snRNA-seq data of in vitro human islets, the β1 cluster in human islets in vivo displayed a similar level of *INS* expression compared with the β2 and β3 clusters (Fig. 7B,C). Similar results were observed when a previous snRNA-seq dataset from human islet grafts [22] was analyzed (Additional file 1: Fig. S3D-E). In addition, in our dataset, the expression of *ZNF385D* and *HNRNPA2B1* appeared to be similar in the β-cell sub-clusters of human islets in vivo and in vitro (Fig. 7D–G). Importantly, the proportion of cells in the β1 sub-cluster with stable *INS* expression was significantly increased while the proportion of cells in the β2 transition cluster and the β3 with mostly *INS* pre-mRNA were significantly decreased in human islets in vivo compared with in vitro (Fig. 7H). We speculate that this may represent a phenotypic or maturational transfer of cells from the lower *INS* expressing β3 and β2 groups to the more mature *INS* mRNA β1 group, potentially reflecting maturational features of the in vivo microenvironment as compared to the less physiological in vitro conditions.

**Fig. 8:**
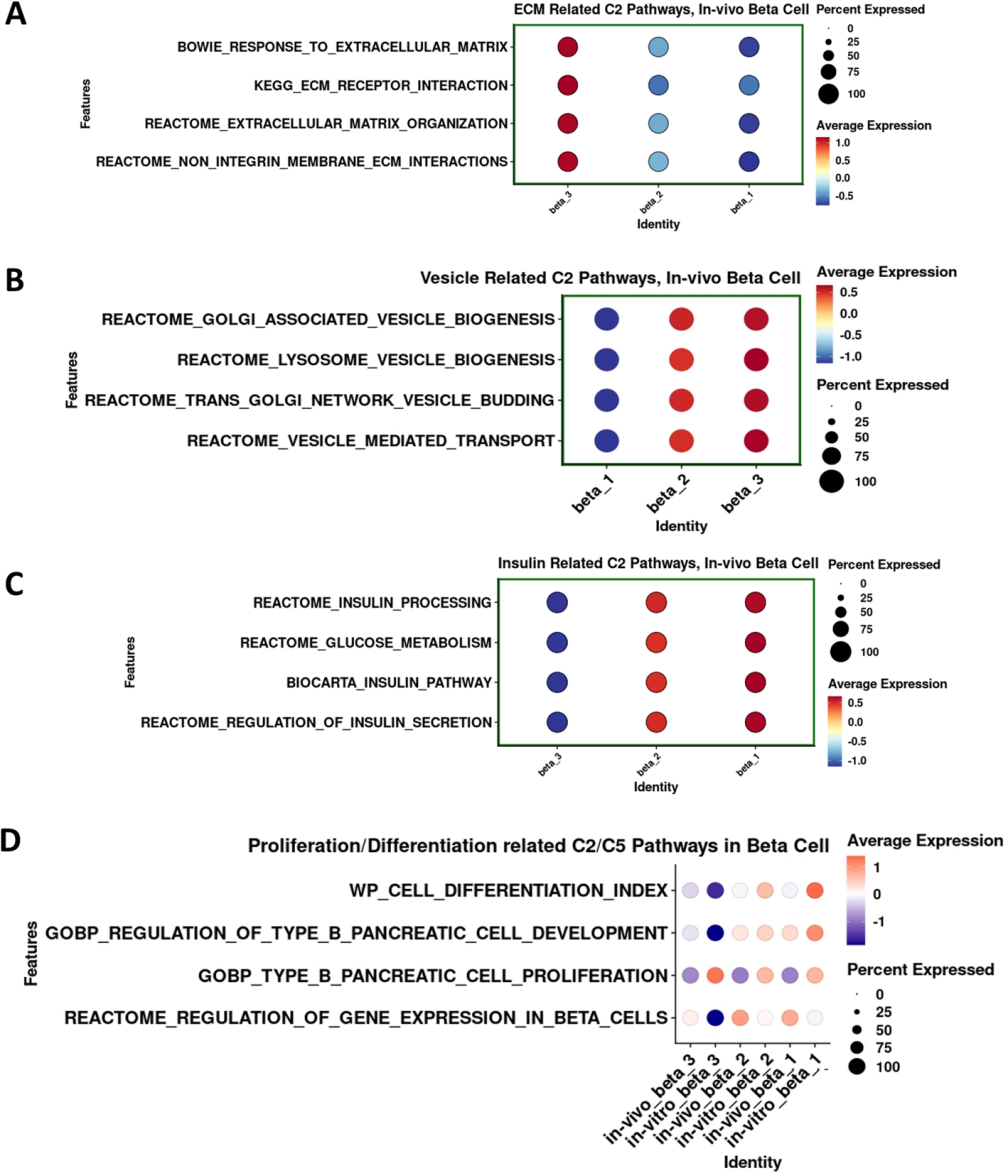
Cellular processes identified by GSEA of the three β-cell sub-clusters β1, β2, and β3 in the in vivo snRNA-seq compared with the in vitro dataset. Dotplot depicting the expression levels and percentage of cells expressing four representatives. **A** ECM-related C2 pathway genes (interaction, response, organization, and integrins), **B** vesicle-related C2 pathway genes (biogenesis, budding, transport, lysosome), **C** insulin-related C2 pathway genes (processing, secretion and glucose metabolism), and **D** proliferation/differentiation related C2 pathway genes (proliferation, development, differentiation, regulation of gene expression) in the in vivo snRNA-seq compared with the in vitro dataset

We also performed gene set enrichment analysis of the different β-cell subpopulations of human islets in vivo using the same approach as above (Figs. 5 and 8). As observed in vitro (Fig. 5), the β3 cell subtype displayed higher expression levels of genes involved in ECM biological processes compared with β2 and β1 cell subtypes (Fig. 8A). The expression of genes involved in the biological processes of intracellular vesicle budding, transport and formation, insulin secretion, processing and glucose metabolism, β-cell differentiation, development, proliferation, and gene expression regulation in the β2 and β1 cell subtypes remained comparable in vivo and in vitro (Fig. 8B–D). Interestingly, as shown in Additional file 1: Fig. S7, the expression of genes included in the “reactome_glucose-metabolism” gene set such as *SLC2A2*, *GK*, *PFKFB2*, and *GPI* increased their expression while *G6PC2* decreased expression in β2 and β1 compared with β3 cells suggesting upregulation of glycolysis-related gene pathways. This correlates with β2 and β1 displaying higher expression of genes involved in differentiation/maturity and insulin secretion. Indeed, *HKI* and *HKIII*, *SLC2A1* and *SLC2A3*, *LDHA*, *ALDOB* and *SLC16A1* (“disallowed” genes, [52] were expressed at very low levels or not expressed at all in the β-cell sub-clusters. Notably, human β3 cells displayed lower expression of genes involved in β-cell proliferation in vivo than in vitro (Fig. 8B–D), suggesting again perhaps that the in vivo microenvironment may provide cues for β-cells to favor a more functional, but less mitogenic status compared with the less physiologic in vitro setting.

## Discussion

Over the past decade, scRNA-seq analysis has been employed to analyze specific human islet cell populations and their transcriptome profiles in vitro. However, its application for analyzing human islet cell populations in pancreas samples postmortem or islet grafts post-transplantation has been limited by the need for mechanical and/or enzymatic tissue cell dissociation and inadequate low yields of cell numbers that likely reflect cells that were resistant to damage and death and are also removed from their normal cell–cell interactions [18–20, 46, 53, 54]. In addition, in many cases, only frozen or fixed human islets/pancreas samples, that are unsuitable for preparation of single cells, are available. These limitations complicate the use of scRNA-seq for in vivo human pancreas/islet tissue analysis. In this report, we make eight important new observations that address these problems.

First, we demonstrate for the first time that snRNA-seq can accurately annotate human islet cell types in vivo, their transcriptomic profiles, their heterogeneity, and their corresponding biological processes. Second, we make the observation that including intronic pre-mRNA reads in the snRNA-seq analysis provides greater accuracy and superior correlation scores than using only exonic reads for human islet cell type annotation. Third, we provide the first reference annotation library (coding- or non-coding region-based) for human islet snRNA-seq. Remarkably, this annotation library proves to be superior in unsupervised cell cluster annotation to existing “canonical” gene set annotation markers for snRNA-seq. Fourth, to our surprise, the top four differentially and selectively expressed genes in human endocrine cells derived from snRNA-seq are not the “canonical genes” used to define islet cell populations in established gene sets. Fifth, through integration of scRNA-seq and snRNA-seq datasets in the same human islets, and by comparing the expression of both mature mRNA and pre-mRNA encoded by the *INS* gene, the principal β-cell identity factor, we detect three different β-cell subpopulations with different transcriptome profiles, ranging from an *INS* pre-mRNA-rich cluster to a cluster rich in mature *INS* mRNA. Sixth, using GSEA tools, we observe that the three β-cell subtypes likely perform different fundamental biological processes. Seventh, we extend these integrated datasets from in vitro scRNA-seq and snRNA-seq studies to in vivo in human islet grafts. We observe these same global islet cell populations and the three apparently distinct β-cell subpopulations, validating the use of snRNA-seq on intact, un-dissociated human islets, and on intact human islet grafts. These approaches can also be applied to stored intact tissues as well [21]. And eighth, we find that the proportions of cells in the three β-cell clusters in vivo are altered as compared to in vitro, in agreement with the notion that the in vivo environment differs from in vitro culture conditions, in a manner that favors a mature, fully functional β-cell phenotype (β1 cluster) over an incompletely differentiated proliferative β-cell phenotype (β3 cluster). Collectively, these observations provide a new lens through which to understand human islet cell biology, and new tools to explore human islet cell biology in vivo.

Our initial goal was to establish a method that could be leveraged to study intact human islet cell populations in human islet grafts in mice and defining their specific transcriptome profile in vivo. The current reference libraries to define human pancreas cell populations and transcriptome profiles have been derived from scRNA-seq studies using principally in vitro studies based on exon reads, with the rationale that mature RNAs comprise the majority of cellular mRNA in intact cells [7, 28–32]. However, the majority of reads obtained from our snRNA-seq analysis relate to intron sequences, reflecting the predominance of pre-mRNA in the nucleus. Based on this, we first explored whether the snRNA-seq dataset with and without intronic reads would provide similar results when projected onto the already established Azimuth scRNA-seq human pancreas reference library [7, 28–32]. Unexpectedly, the inclusion of intron reads in the snRNA-seq dataset actually enhanced the prediction score for cell type annotation in the Azimuth reference. Similar results have been found in other tissues as well [24]. Thus, we elected to incorporate intron plus exon reads in our subsequent analyses.

We were surprised to observe that expression of the main “canonical” genes used to define human endocrine cell populations in scRNA-seq analysis (*INS*, *GCG*, *SST*, and *PPY*) were not the top four differentially and selectively expressed genes in the snRNA-seq studies. This indicates that the most abundant mRNAs in human islet endocrine cells do not reflect the most profused pre-mRNAs in those cells. This is perhaps not surprising when considering that a large proportion of steady-state cytoplasmic mRNA in β-cells is the relatively stable insulin mRNA, stored on polyribosomes, awaiting translation in response to a glucose stimulus [55]. Eukaryotic gene expression is controlled by multiple steps: first, pre-mRNA is transcribed in the nucleus; second, pre-mRNA is spliced; third, mature mRNA is assembled with specific RNA-binding proteins forming a messenger ribonucleoprotein (mRNP) complex; fourth, mRNPs are targeted to and translocate through nuclear pore complexes (NPCs) in the nuclear membrane; and fifth, the mRNPs are directionally released into the cytoplasm for translation [56–59]. Alterations in any of these steps can lead to changes in the nuclear content of specific pre-mRNAs and in gene expression patterns [58, 59]. Insulin pre-mRNA contains two introns that are not spliced with the same efficiency [60, 61]. In human islets, intron 1– and intron 2–containing *INS* pre-mRNAs are ~ 150- and 2000-fold less abundant, respectively, than mature *INS* mRNA [61]. Importantly, it has been suggested that *INS* pre-mRNA levels (particularly intron 2–containing pre-mRNAs) may be the most reliable reflection of acute changes to human *INS* gene transcription induced by glucose [61]. Based on these studies, and since our experiments compared scRNA-seq and snRNA-seq in the same human samples cultured at 5 mM glucose, the fact that *INS* mRNA is the top gene in scRNA-seq but not in the snRNA-seq suggests that this is not the result of changes in glucose stimulus, donor demographics, splicing rates, mRNPs, NPC complexes, or translation rates. Nevertheless, these are parameters that need further studies to decipher how they can alter *INS* and other pre-mRNAs in multiple conditions affecting endocrine cells including aging, hyperglycemia, and inflammation among others. These findings also mandate development of new gene sets and a new reference library for annotation of snRNA-seq datasets derived from human islet samples. Here, we have established this reference library and identified new gene sets for human endocrine cells using snRNA-seq analysis. These include *ZNF385D*, *TRPM3*, *LRFN2* and *PLUT* (for β-cells); *PTPRT*, *FAP*, *PDK4*, and *LOXL4* (for α-cells); *LRFN5*, *ADARB2*, *ERBB4*, and *KCNT2* (for δ-cells); and *CACNA2D3*, *THSD7A*, *CNTNAP5*, and *RBFOX3* (for γ-cells). Analysis of the only published data on snRNA-seq in isolated human islets [22] confirmed the usefulness of this new gene sets for snRNA-seq analysis using exon + intron reads. To validate *ZNF385D*, encoding zinc finger protein 385D, involved in neurocognitive development in the brain [62], but whose presence and function have not been demonstrated in human β-cells thus far, we performed *ZNF385D* RNA scope. We show for the first time that an intronic *ZNF385D* probe detects the gene in nuclei, while an exonic *ZNF385D* probe detects the gene in both nuclei and cytoplasm of human β-cells, but not other islet cell types. Future studies will need to decipher the biological role of this zinc finger protein in the human β-cell.

Through integration of the scRNA-seq and the snRNA-seq datasets from human islets in vitro, and using *INS* expression as the starting point in the pseudo-time and RNA velocity analyses, we identify three distinct human β-cell subpopulations. Since the 1980s, heterogeneity of rodent and human β-cells has been described identifying β-cell subpopulations based on their different capacities of insulin secretion, insulin biosynthesis, glucose metabolism, Ca2 + dynamics, intracellular network connectivity, membrane excitability and electrical coupling, and hub cells, leader cells, first responders, wave originators, and followers have been identified among the different β-cells [63–68]. β-cell heterogeneity can influence when diabetes occurs and the response to treatments; therefore, identification of functional and regenerative characteristics and markers of distinct β-cell subpopulations can help in the design of novel regenerative and functional approaches to treat diabetes [69]. Studies by Lickert’s group have identified two subpopulations of β-cells, proliferation-competent β-cells, and mature β-cells with distinct molecular, physiological, and ultrastructural features [70]. scRNA-seq approaches [16, 17, 70] have also identified several human β-cell subpopulations with different transcriptome profiles, but never using a combination of both scRNA-seq and snRNA-seq techniques. Indeed, scRNA-seq studies have identified different human β-cell subtypes based on their transcriptional profiles on ER stress and autophagy markers and functional profiles [16, 17, 71]. Transcriptional deregulation leading to cellular immaturity, reorganization of β-cell transcription factor networks, and reduced glucose-stimulated insulin release in different β-cell subtypes occurs during β-cell aging [72]. In the current study, and since *INS* mRNA expression is higher in β3 cells in the snRNA-seq data, we postulate that β3 cells represent the β-cells which have higher levels of *INS* pre-mRNA. Conversely, in β1 cells where there is little or no expression of *INS* in the snRNA-seq, but very high expression level in scRNA-seq, we speculate that these β-cells represent a population in which mature *INS* mRNA is stored, awaiting transcription. Finally, β2 cells appear to represent cells in a transitional stage closer to β1. In support of these possibilities, GSEA analysis of the biological processes occurring in these β-cell sub-clusters highlighted that although all are β-cells, β1, β2, and β3 display a spectrum of functions with respect to ECM, vesicle formation, insulin processing and secretion, glucose metabolism, proliferation, gene expression regulation, and differentiation. Comparison analysis with previous β-cell subpopulations identified by Dorrell et al. [16] shows that at least with the CD9 cell marker, the current study’s β3 subtype correlates with Dorrell et al. β2/β4 and the current study’s β1 (highest expression in genes related to regulation of insulin secretion) to β1/β3 (highest insulin secretion response) in the Dorrell et al. report [16]. This illustrates the utility of snRNA-seq analysis for further deciphering the heterogeneity in defined β-cell populations. However, the lack of functionality tests in the human islet cells limits the association of β-cell subpopulations with their functional state in our studies.

Recently, Reddick et al. have analyzed human islet grafts using scRNA-seq and identified populations of α-, β-, and δ-cell subsets [20]. These studies used a large number of islets (4000) transplanted into mice and required enzymatic and mechanical tissue dissociation of the islet grafts. Assuming an average of 1000 cells per islet, 4000 islets should contain something in the range of 4,000,000 cells. Importantly, Reddick et al. retrieved approximately 700 cells for analysis. This is a general experience and further emphasizes the concept that the small subset of cells recovered from intact tissue may represent the few most able to resist harsh cell harvesting techniques, and not the general population of islet cells.

More recently, Basile et al. also reported the use of snRNA-seq on human islet grafts transplanted into mice [22]. This study excluded intronic reads, focusing only on exonic reads, and observed a strong concordance among islet cell types and gene signatures between cells analyzed by scRNA-seq and snRNA-seq. In retrospect, we speculate that this study likely would have been further enhanced by including intronic reads, these being the most abundant nuclear reads, both with respect to accuracy of islet cell type annotation, and gaining additional information on the differential pre-mRNA levels of genes of interest. This is illustrated here, where we found that the novel gene annotation data derived from the snRNA-seq analysis in vitro permitted us to define the identity of the human islet cells in the islet grafts with greater accuracy and resolution than scRNA-seq, and also allowed us to distinguish the pre-mRNA status of cells and genes of interest. Using this approach, we were able to identify the three β-cell subpopulations in grafts in vivo enriched for similar biological processes as those observed in vitro. Furthermore, these results were also confirmed when using the human islet dataset from the Basile et al. paper [22]. Interestingly, by pseudo-time analysis, we observed that cells in clusters β3 and β2 appeared to transition into, or at least be replaced by β1 cells, as islets moved from in vitro to in vivo, such that β1 cells became the most prominent β-cell subtype in the human islet graft. Nevertheless, the change in the proportion of β-cell sub-clusters found between islets and islet grafts could also reflect the different organization of the endocrine tissue in both conditions and not related to the response of β-cells to the in vivo environment per se. Future studies employing spatial transcriptomics which provide morphological context to gene expression changes and presence of different cell subclasses are warranted to determine this possibility.

Interestingly, although the “biological processes” categorizations were generally maintained in the three β-cell sub-clusters in vitro and in vivo, we note that the “proliferation” biological process was present in the β3 sub-cluster in vitro, but not present in this sub-cluster in vivo. This makes several points. First, β-cell proliferative capacity is typically higher in in vitro studies as compared to in vivo studies. This dichotomy is clearly illustrated in studies testing therapeutic agents that induce β-cell proliferation in vivo for regenerative purposes, which consistently reveal the lower β-cell proliferation rates in vivo than in vitro [26, 27, 71]. Second, dynamic changes occur among the different β-cell subpopulations in vitro and in vivo. And third, that there is an inverse correlation between the β-cell proliferation process and insulin secretion at least in vitro since β3 cells have proliferative capacity, but low insulin secretion pathway, while in β1 cells it is the opposite, highlighting the proliferation-function dichotomy in β-cells [73, 74]. Finally, the use of snRNA-seq and β-cell subpopulations analysis in vivo in islet grafts invites a future plethora of studies analyzing the effect of drugs and physiological and pathological situations in vivo in human islet cells. Furthermore, harnessing the power of this technique that allows capturing of cell classes that are difficult to achieve by dissociation based scRNA-seq including endothelial cells, resident macrophages among others could help to identify and analyze rare cell subpopulations and their transcriptomic profiles.

In summary, the current limitations to obtain a high yield of unstressed cells from tissues for sc-RNA-seq analysis together with the information in this study provide strong support for the use of snRNA-seq analysis and intron-inclusive libraries to define cell populations and transcriptome profiles of islet cells in human islets/pancreas tissue in vivo. Using this approach, we have found new gene sets to define islet cell populations in snRNA-seq studies that can be used for islet cell identification and transcriptome profile in vivo. We have identified three β-cell subpopulations with dynamic gene profiles under basal conditions. Finally, we show that that this technique will be valuable in assessing effects on human islet cell subtypes in islet grafts transplanted into mice in response to multiple physiologic, pathologic, and therapeutic challenges, as well as models of T1D and T2D.

## Conclusions

We propose that snRNA-seq and pre-mRNA analysis can accurately identify human islet cell populations, subpopulations, and their dynamic transcriptome profile in vitro and in vivo in human islet grafts.

## Supplementary Information

**Additional file 1: Table S1.** Characteristics of human islet donors and human islet preparations used for the transcriptomic analysis. **Table S2.** Number of cells/nuclei loaded, targeted, and recovered, and the mean and median of reads and genes per cell/nuclei for the different samples used in this study. **Figure S1.** Gene overlap between scRNA-seq and snRNA-seq using UMIs > 0.1,1,10,20,50,100. **Figure S2.** Analysis of reference alignment prediction score, expression level and percentage of cells using subsampling to the same UMI depth for both intron-inclusive library and the exonic only library. **Figure S3.** snRNA-seq gene markers in human islet cells and β-cell subtypes in the previously published dataset for snRNA-seq data in vitro and in vivo from Basile et al., reference 22. **Figure S4.** Analysis of β-cell inter-cluster 3-2 region using a high Louvain algorithm resolution of 2.0 to resolve smaller clusters. **Figure S5.** RNA Velocity analysis of integrated and individual scRNA-seq and snRNA-seq data from human islets in vitro. **Figure S6.** Differentially expressed gene sets from β-cell subtypes in Dorrell et al. reportprojected into the β-cell subclusters of the current study. **Figure S7.** Gene expression in the “reactome_glucose-metabolism” gene set plus SLC2A1-3, LDHA and SLC16A1genes in the different β-cell subclusters of the current study.

**Additional file 2: Data S1.** Differentially expressed genes in the different human islet cell clusters from the scRNA-seq studies.

**Additional file 3: Data S2.** Differentially expressed genes in the different human islet cell clusters from the snRNA-seq studies in vitro.

**Additional file 4: Data S3.** Differentially expressed genes in the different human β-cell subclusters from the integrated, scRNA-seq and snRNA-seq in vitro studies and the snRNA-seq in vivo studies.

**Additional file 5: Data S4.** Differentially expressed genes in the human β-cell 3-2 transition subclusters from the integrated datasets in the in vitro studies.

**Additional file 6: Data S5.** Differentially expressed genes in the different human islet cell clusters from the snRNA-seq studies in vivo.

## Data Availability

All raw sequencing reads for the scRNA-seq and snRNA-seq data generated from cultured human islets and engrafted human islet tissues are compiled and available from NCBI SRA PRJNA900882 (https://www.ncbi.nlm.nih.gov/geo/query/acc.cgi?acc=GSE217837) under accession number GSE217837 [75]. The publicly available scRNA-seq and snRNA-seq datasets on human islets that were also used in this manuscript are from Basile G et al. [22] who used single-nucleus RNA sequencing to interrogate transcriptomic profiles of archived human pancreatic islets, PRJNA856476, NCBI Sequence Read Archive, https://www.ncbi.nlm.nih.gov/bioproject/PRJNA856476 and PRJNA631512, NCBI Sequence Read Archive, https://www.ncbi.nlm.nih.gov/bioproject/PRJNA631512. R/Shell/Python code for data handling and analysis is publicly available through from Github repository (https://github.com/randystyle21/scRNA-snRNA-Marker/blob/main/README.md) [76].
