# Supplementary material for: Single-nucleus RNA sequencing of human pancreatic islets identifies novel gene sets and distinguishes β-cell subpopulations with dynamic transcriptome profiles
Source: Genome Med. 2023 May 1;15:30. doi: 10.1186/s13073-023-01179-2 (PMC10150516; doi:10.1186/s13073-023-01179-2)
Supplement: Supplementary file 1 — Additional file 1: Table S1. Characteristics of human islet donors and human islet preparations used for the transcriptomic analysis. Table S2. Number of cells/nuclei loaded, targeted, and recovered, and the mean and median of reads and genes per cell/nuclei for the different samples used in this study. Figure S1. Gene overlap between scRNA-seq and snRNA-seq using UMIs > 0.1,1,10,20,50,100. Figure S2. Analysis of reference alignment prediction score, expression level and percentage of cells using subsampling to the same UMI depth for both intron-inclusive library and the exonic only library. Figure S3. snRNA-seq gene markers in human islet cells and β-cell subtypes in the previously published dataset for snRNA-seq data in vitro and in vivo from Basile et al., reference 22. Figure S4. Analysis of β-cell inter-cluster 3-2 region using a high Louvain algorithm resolution of 2.0 to resolve smaller clusters. Figure S5. RNA Velocity analysis of integrated and individual scRNA-seq and snRNA-seq data from human islets in vitro. Figure S6. Differentially expressed gene sets from β-cell subtypes in Dorrell et al. reportprojected into the β-cell subclusters of the current study. Figure S7. Gene expression in the “reactome_glucose-metabolism” gene set plus SLC2A1-3, LDHA and SLC16A1genes in the different β-cell subclusters of the current study. [file 13073_2023_1179_MOESM1_ESM.pdf]

**Table S1:**

## Checklist for Reporting Human Islet Preparations Used in Research

Adapted from Hart NJ, Powers AC (2018) Progress, challenges, and suggestions for using human islets to understand islet biology and human diabetes. Diabetologia https://doi.org/10.1007/s00125-018-4772-2

| Islet preparation | 1 | 2 | 3 | 4 | 5 | 6 | 7 |
|---|---|---|---|---|---|---|---|
| | | | | | | | |
| Unique identifier | HP20240 | HP20245 | HP21189 | HP20234 | HP21055 | HP21203 | HP21197 |
| Donor age (years) | 42 | 24 | 26 | 58 | 36 | 25 | 53 |
| Donor sex (M/F) | M | M | M | F | F | M | M |
| Donor BMI (kg/m$^2$) | 23.5 | 20.0 | 26.3 | 26.9 | 31.6 | 26.5 | 32.4 |
| Donor $HbA_{1c}$ or other measure of blood glucose control | 5.4% | 5.4% | 5.4% | 5.5% | 5.6% | 5.8% | 5.5% |
| Origin/source of islets[b] | Prodo Lab | Prodo Lab | Prodo Lab | Prodo Lab | Prodo Lab | Prodo Lab | Prodo Lab |
| Islet isolation centre | Prodo Aliso Viejo, CA | Prodo Aliso Viejo, CA | Prodo Aliso Viejo, CA | Prodo Aliso Viejo, CA | Prodo Aliso Viejo, CA | Prodo Aliso Viejo, CA | Prodo Aliso Viejo, CA |
| Donor history of diabetes? Please select yes/no from drop down list | No | No | No | No | No | No | No |
| | | | | | | | |
| Diabetes duration (years) | | | | | | | |
| Glucose-lowering therapy at time of death[c] | | | | | | | |

*Continues on the next page*

| Donor cause of death | Stroke | Head trauma | Head trauma | Stroke | Stroke | Head trauma | Anoxic event |
|---|---|---|---|---|---|---|---|
| Warm ischaemia time (h) | N/A | N/A | N/A | N/A | N/A | N/A | N/A |
| Cold ischaemia time (h) | N/A | N/A | N/A | N/A | N/A | N/A | N/A |
| Estimated purity (%) | 95 | 95 | 90 | 90 | 90 | 95 | 90 |
| Estimated viability (%) | 95 | 95 | 95 | 95 | 95 | 95 | 95 |
| Total culture time (h)[d] | 120 | 120 | 120 | 96 | 96 | 144 | 120 |
| Glucose-stimulated insulin secretion or other functional measurement[e] | N/A | N/A | N/A | N/A | N/A | N/A | N/A |
| Handpicked to purity? Please select yes/no from drop down list | Yes | Yes | Yes | Yes | Yes | Yes | Yes |
| Additional notes | | | | | | | |

[a]If you have used more than eight islet preparations, please complete additional forms as necessary
[b]For example, IIDP, ECIT, Alberta IsletCore
[c]Please specify the therapy/therapies
[d]Time of islet culture at the isolation centre, during shipment and at the receiving laboratory
[e]Please specify the test and the results
N/A Not available

**Table S2. Information on cell Ranger processed data prior to quality control.**

| Donor Code | Processing | Cells/Nuclei Loaded | Cells/Nuclei Target | Cells/Nuclei Recovered | Mean Reads per Cell/Nuclei | Median Genes per Cell/Nucleus |
|---|---|---|---|---|---|---|
| HP20240-01 | scRNA_1 | 16,000 | 10,000 | 8,448 | 19,821 | 1,932 |
| HP20240-01 | snRNA_1 | 13,000 | 8,000 | 6,737 | 27,984 | 2,083 |
| HP20245-01 | scRNA_2 | 10,000 | 6,000 | 5,542 | 53,847 | 2,570 |
| HP20245-01 | snRNA_2 | 8,000 | 5,000 | 3,950 | 96,741 | 1,119 |
| HP21189-01 | scRNA_3 | 16,000 | 10,000 | 8,627 | 43,017 | 2,405 |
| HP21189-01 | snRNA_3 | 5,000 | 3,000 | 1,780 | 121,247 | 1,880 |
| HP20234-01 | Islet graft snRNA 1 | 10,000 | 6,000 | 4,599 | 77,548 | 1,848 |
| HP21055-01 | Islet graft snRNA 2 | 6,000 | 4,000 | 2,796 | 109,167 | 1,537 |
| HP21203-01 | Islet graft snRNA 3 | 9,000 | 6,000 | 5,021 | 68,527 | 2,601 |
| HP21197-01 | Islet graft snRNA 4 | 3,000 | 2,000 | 909 | 349,619 | 2,945 |

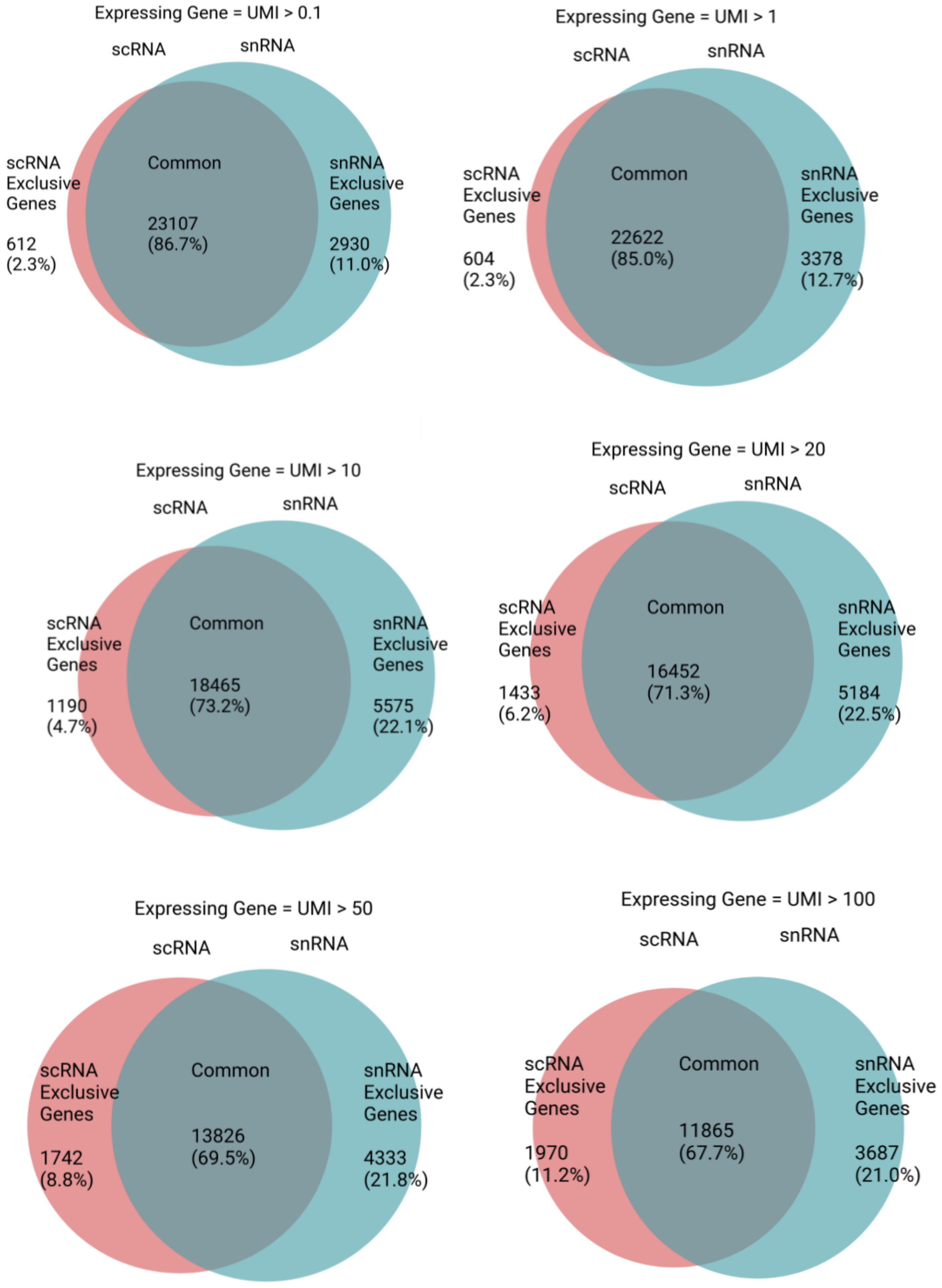


**Fig. S1: Gene Overlap Between scRNA-seq and snRNA-seq.** Venn diagrams of genes detected in both scRNA-seq and snRNA-seq analysis of the three human islet samples using UMIs > 0.1,1,10,20,50,100.

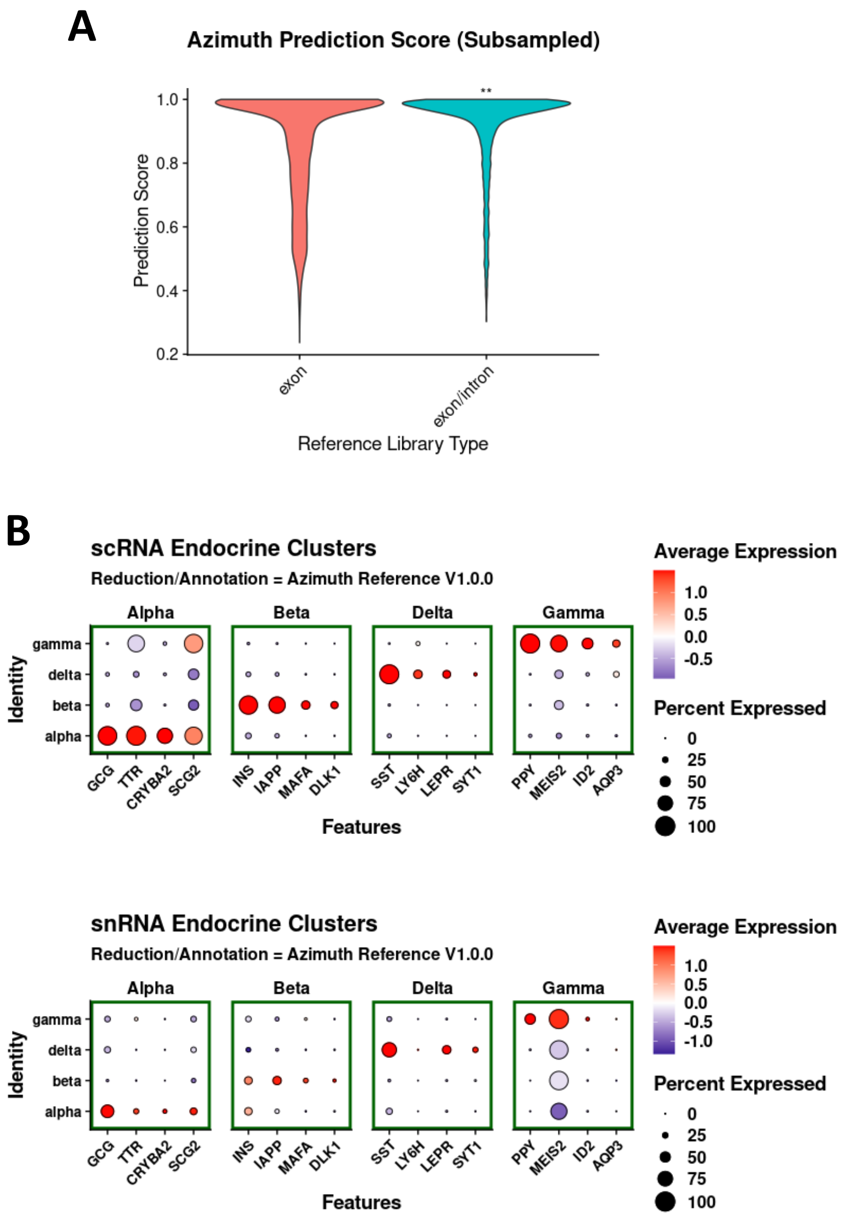


**<u>Fig. S2:</u> Analysis of Reference Alignment Prediction Score, Expression Level and Percentage of Cells Using Subsampling to the Same UMI Depth. A.** Prediction score in the snRNA-seq analysis with only exon reads or with both exon and intron reads using subsampling. We subsampled the intron-inclusive library to the median value of 1247 UMIs that is the median of exonic only library. Statistical significance was tested using a Wilcoxon rank-sum test. **B.** Expression level and percentage of cells expressing canonical genes of Azimuth-annotated endocrine cell types in the scRNA-seq data (top) and snRNA-seq data (bottom) by subsampling the UMI to median of snRNA sequencing depicted in Fig. 1C.

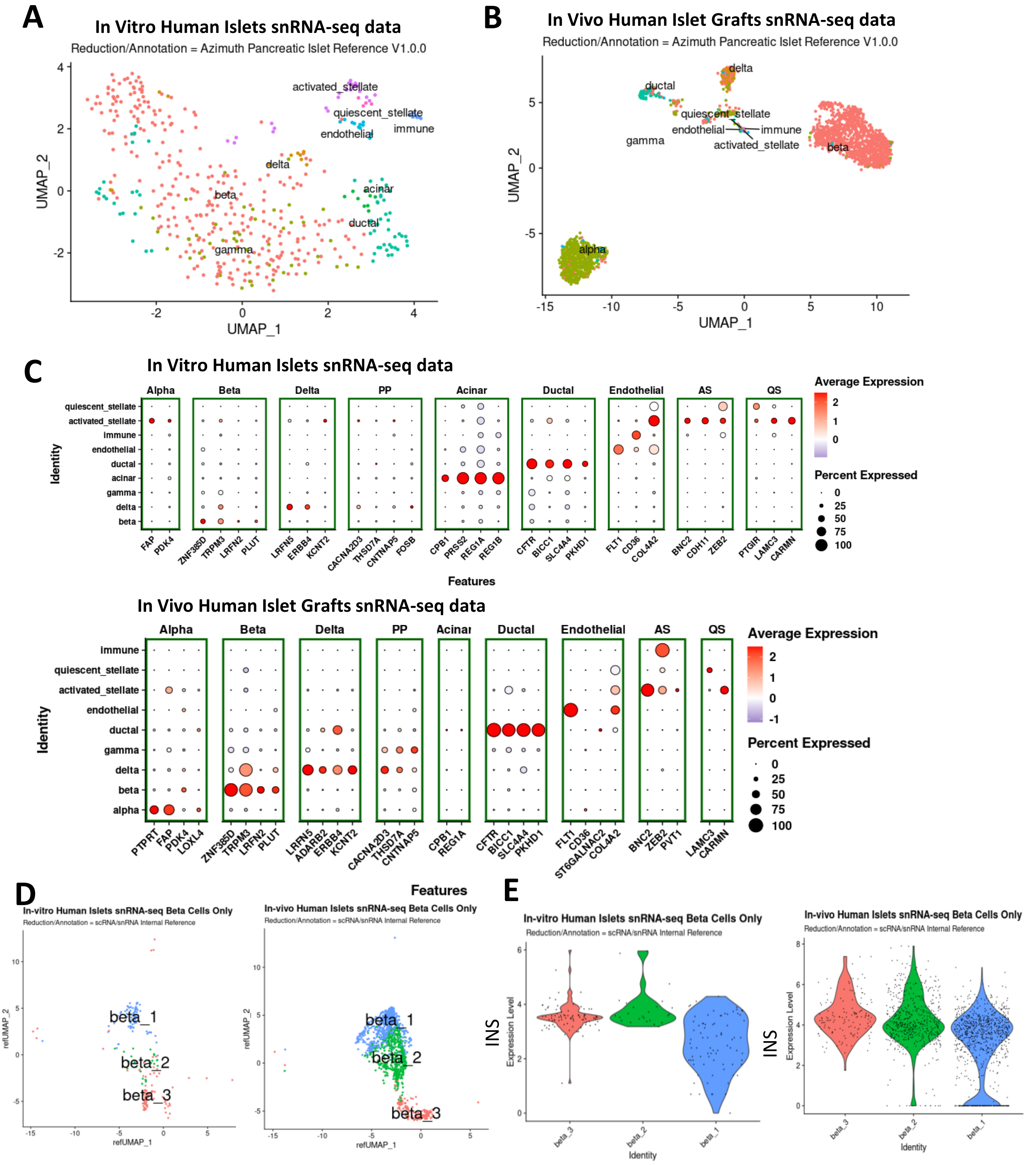


**Fig. S3: snRNA-seq Gene Markers in Human Islet Cells and β-Cell Subtypes in a Previously Published Dataset. A.** Human islet cell type identification by projection strategy using Azimuth pancreatic islet reference v1.0.1 and intron+exon library of the dataset from ref. 22. Due to the limited number of cells, we could not project UMAP reduction successfully, thus showing the unsupervised clustering instead. **B.** Projection of the human islet graft dataset from the same publication. **C.** Dotplot projection of newly found gene sets on Azimuth annotated endocrine clusters for snRNA-seq data *in vitro* and *in vivo* from ref. 22. **D.** Identification of β-cell clusters using the snRNA-seq dataset from ref. 22 using the internal scRNA-seq/snRNA-seq of the current study. **E.** *INS* expression level on violin plots in the three β-cell subtypes in the published *in vitro* and *in vivo* snRNA-seq data from ref. 22.

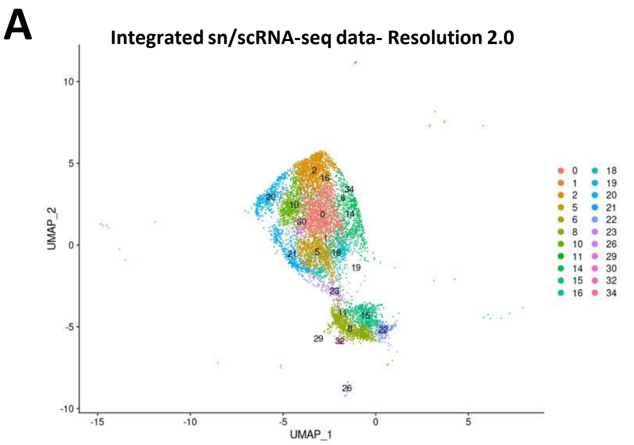


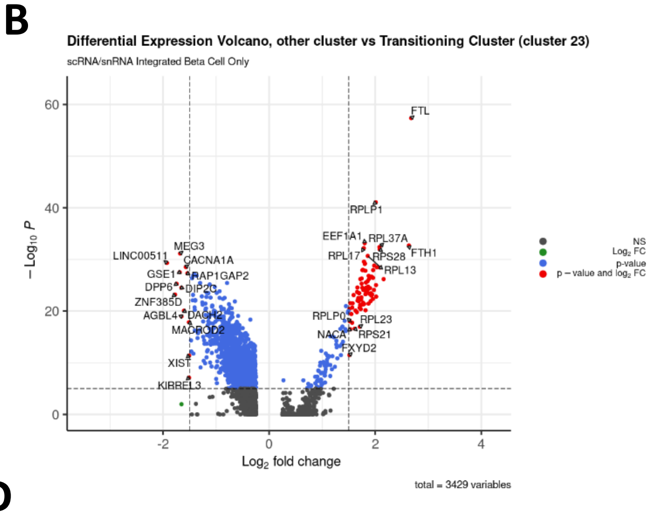


C

| gene | gene_biotype | description |
|---|---|---|
| FTL | protein_coding | ferritin light chain |
| FTH1 | protein_coding | ferritin heavy chain 1 |
| RPL34 | protein_coding | ribosomal protein L34 |
| RPL41 | protein_coding | ribosomal protein L41 |
| RPS28 | protein_coding | ribosomal protein S28 |
| RPL37A | protein_coding | ribosomal protein L37a |
| RPS8 | protein_coding | ribosomal protein S8 |
| RPS27 | protein_coding | ribosomal protein S27 |
| RPS27 | LRG_gene | ribosomal protein S27 |
| RPL32 | protein_coding | ribosomal protein L32 |
| RPLP1 | protein_coding | ribosomal protein lateral stalk subunit P1 |
| RPL21 | protein_coding | ribosomal protein L21 |
| RPS12 | protein_coding | ribosomal protein S12 |
| RPL23A | protein_coding | ribosomal protein L23a |
| RPS15 | protein_coding | ribosomal protein S15 |
| RPL12 | protein_coding | ribosomal protein L12 |
| TPT1 | protein_coding | tumor protein, translationally-controlled 1 |
| RPL30 | protein_coding | ribosomal protein L30 |
| RPL37 | protein_coding | ribosomal protein L37 |
| RPL31 | protein_coding | ribosomal protein L31 |
| RPL26 | protein_coding | ribosomal protein L26 |
| RPL26 | LRG_gene | ribosomal protein L26 |
| RPS23 | protein_coding | ribosomal protein S23 |
| RPS3A | protein_coding | ribosomal protein S3A |

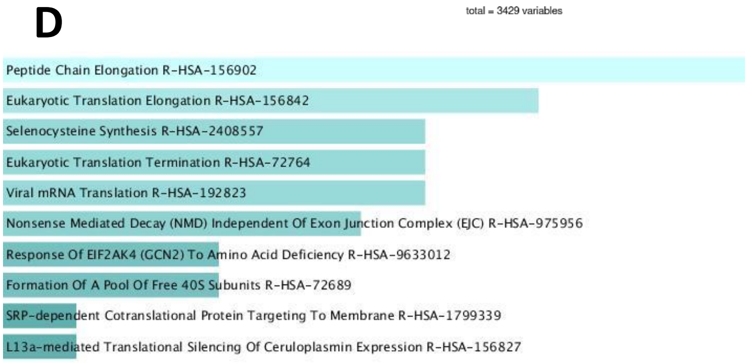


**Fig. S4: Analysis of β-Cell Inter-cluster 3-2 Region. A.** β-cell cluster separation was performed with a high Louvain algorithm resolution of 2.0 to resolve smaller clusters and cluster 23 in between β3 and β2 was identified. **B.** Volcano plot depicting the differentially expressed genes in cluster 23 against all remaining clusters. **C.** Table showing the 25 upregulated genes in cluster 23, mostly ribosomal protein genes. **D.** GSEA (Reactome) depicts translation elongation and termination as the main biological processes in these inter β-3-2 cells.

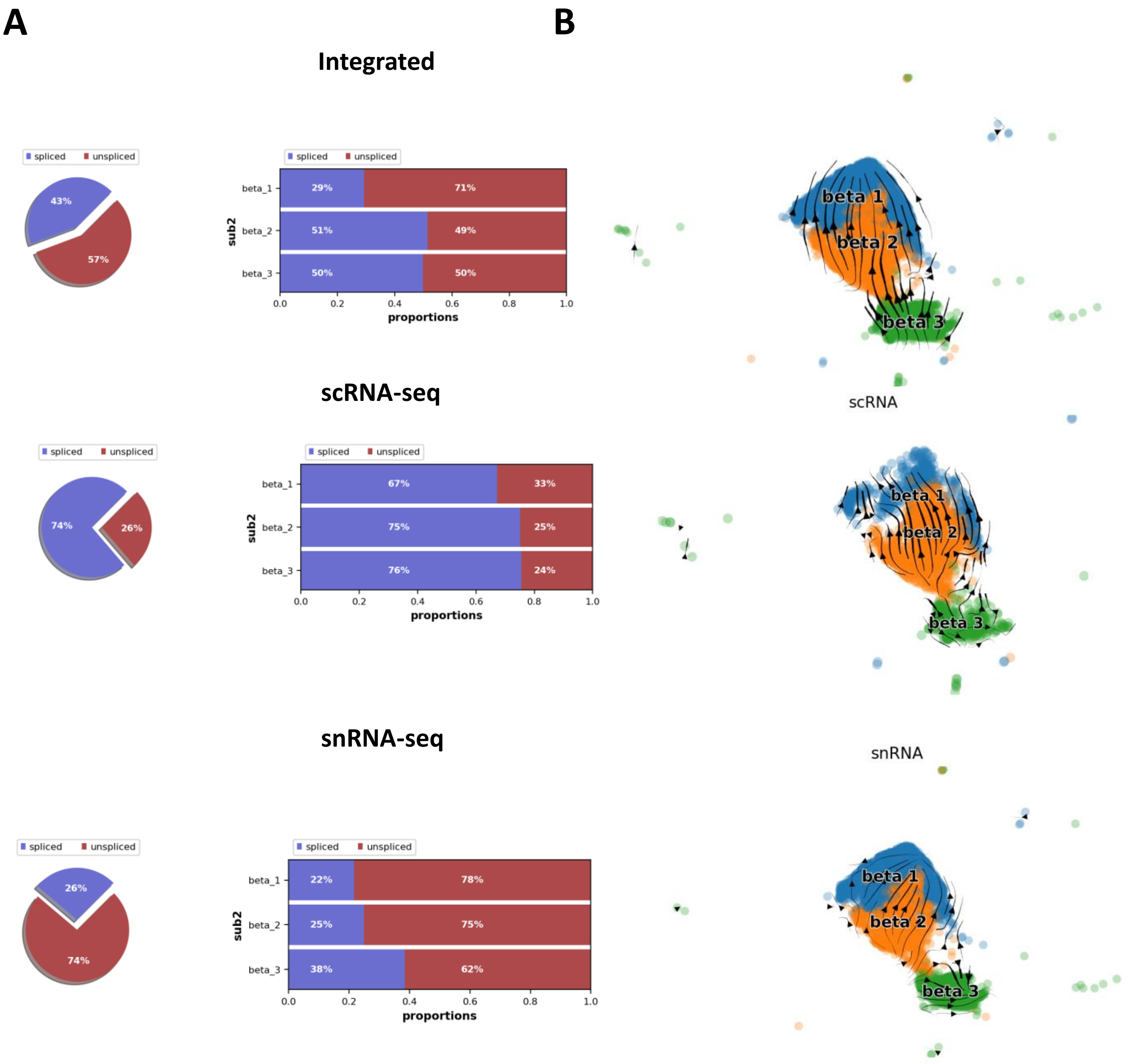


**Fig. S5: RNA Velocity Analysis of Integrated and Individual scRNA-seq and snRNA-seq Data from Human Islets *In Vitro*. A.** Spliced and unspliced count ratios are estimated by separately counting reads that incorporate intronic sequences. Bars show percentage of spliced and unspliced molecules in integrated, scRNA-seq and snRNA-seq datasets. **B.** RNA velocity streamline was estimated with the integrated, scRNA-seq and snRNA-seq datasets. The observed and the extrapolated future (arrows) states are shown without cell or gene pooling.

**A** Genes differentially expressed between the ST8SIA1⁺ β3/β4 and ST8SIA1⁻ β1/β2 subtypes.

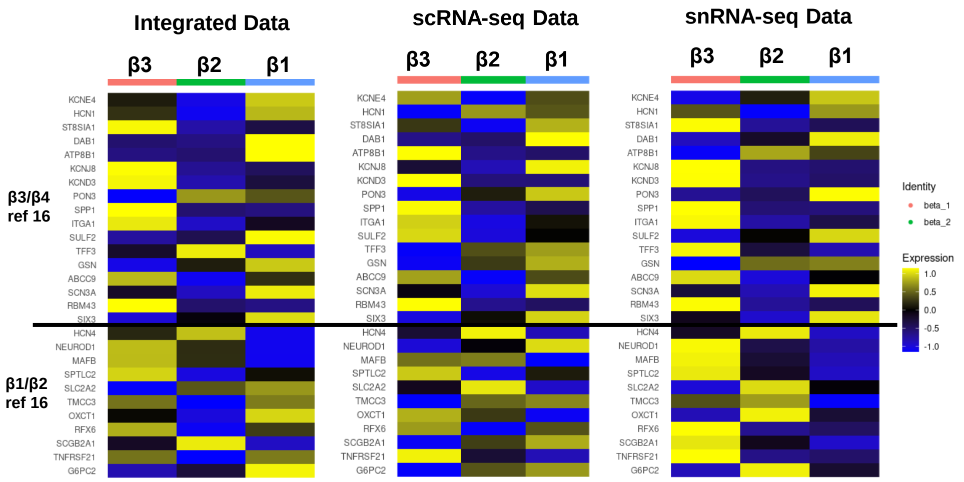


**B** Genes differentially expressed between the CD9⁺ β2/β4 and CD9⁻ β1/β3 subtypes in reference 16.

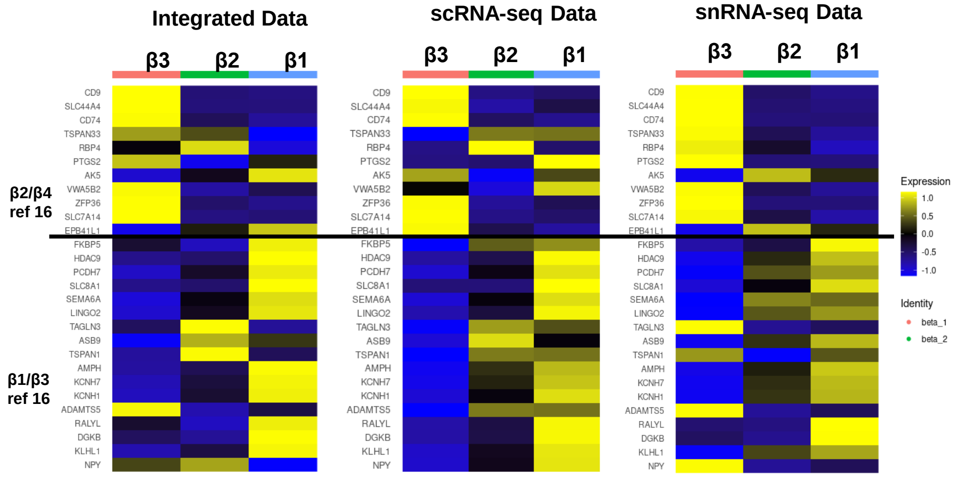


**<u>Fig. S6</u>: Average Heatmap of Differentially Expressed Gene Sets from β-cell Subtypes in Dorrell et al. Report (16) Projected into the β-Cell Subclusters of the Current Study.** Heatmap of differentially expressed genes in β-cell subtypes (β1-3) with integrated scRNA/snRNA-seq, scRNA-seq, and snRNA-seq data compared with **A.** ST8SIA1⁺ (from *HCN4* to *G6PC2*) and ST8SIA1⁻ (from *KCNE4* to *SIX*) cells (β1-4) and **B.** CD9⁺ (from *CD9* to *EPB41L1*) and CD9⁻ (from *FKBP5* to *NPY*) cells (β1-4) according to Fig. 4 in ref. 16.

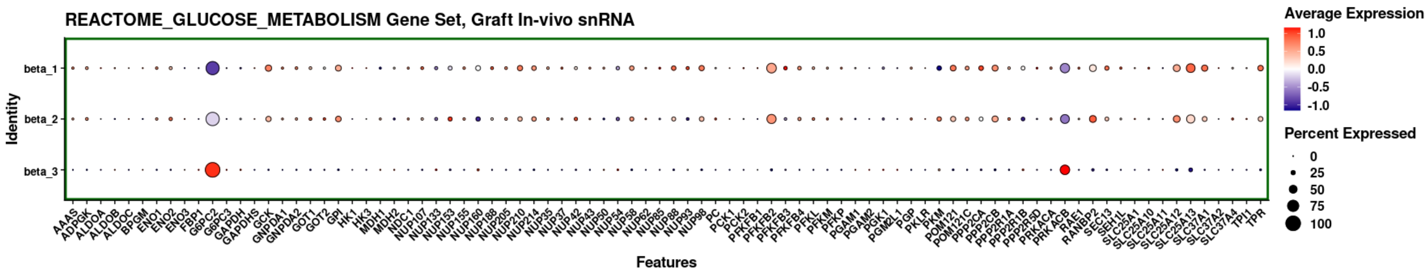


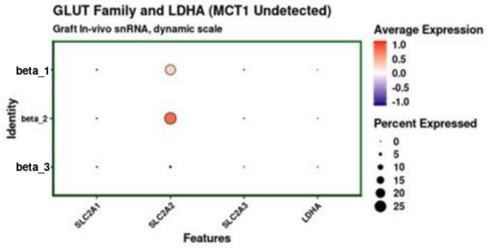


<u>**Fig. S7**</u>**:** Dotplot representing the expression of genes included in the "reactome_glucose-metabolism" gene set (publicly available at https://www.gsea-msigdb.org/gsea/msigdb/cards/REACTOME_GLUCOSE_METABOLISM) plus manually added *SLC2A1-3, LDHA, SLC16A1* genes which are not included in this gene set panel.
